# Insights into genetic variants associated with NASH-fibrosis from metabolite profiling

**DOI:** 10.1093/hmg/ddaa162

**Published:** 2020-07-28

**Authors:** Jake P Mann, Maik Pietzner, Laura B Wittemans, Emmanuela De Lucia Rolfe, Nicola D Kerrison, Fumiaki Imamura, Nita G Forouhi, Eric Fauman, Michael E Allison, Jules L Griffin, Albert Koulman, Nicholas J Wareham, Claudia Langenberg

**Affiliations:** MRC Epidemiology Unit, Institute of Metabolic Science, University of Cambridge, Cambridge CB2 0SL, UK; MRC Epidemiology Unit, Institute of Metabolic Science, University of Cambridge, Cambridge CB2 0SL, UK; MRC Epidemiology Unit, Institute of Metabolic Science, University of Cambridge, Cambridge CB2 0SL, UK; MRC Epidemiology Unit, Institute of Metabolic Science, University of Cambridge, Cambridge CB2 0SL, UK; MRC Epidemiology Unit, Institute of Metabolic Science, University of Cambridge, Cambridge CB2 0SL, UK; MRC Epidemiology Unit, Institute of Metabolic Science, University of Cambridge, Cambridge CB2 0SL, UK; MRC Epidemiology Unit, Institute of Metabolic Science, University of Cambridge, Cambridge CB2 0SL, UK; Internal Medicine Research Unit, Pfizer Worldwide Research, Development and Medical, Cambridge, MA 02142, USA; Liver Unit, Department of Medicine, Cambridge Biomedical Research Centre, Cambridge University Hospitals NHS Foundation Trust, Cambridge CB2 0QQ, UK; MRC Human Nutrition Research, University of Cambridge, Cambridge CB1 9NL, UK; Department of Biochemistry, Cambridge Systems Biology Centre, University of Cambridge, Cambridge CB2 1GA, UK; MRC Human Nutrition Research, University of Cambridge, Cambridge CB1 9NL, UK; Department of Biochemistry, Cambridge Systems Biology Centre, University of Cambridge, Cambridge CB2 1GA, UK; MRC Epidemiology Unit, Institute of Metabolic Science, University of Cambridge, Cambridge CB2 0SL, UK; MRC Epidemiology Unit, Institute of Metabolic Science, University of Cambridge, Cambridge CB2 0SL, UK

## Abstract

Several genetic discoveries robustly implicate five single-nucleotide variants in the progression of non-alcoholic fatty liver disease to non-alcoholic steatohepatitis and fibrosis (NASH-fibrosis), including a recently identified variant in *MTARC1*. To better understand these variants as potential therapeutic targets, we aimed to characterize their impact on metabolism using comprehensive metabolomics data from two population-based studies. A total of 9135 participants from the Fenland study and 9902 participants from the EPIC-Norfolk cohort were included in the study. We identified individuals with risk alleles associated with NASH-fibrosis: rs738409C>G in *PNPLA3*, rs58542926C>T in *TM6SF2*, rs641738C>T near *MBOAT7*, rs72613567TA>T in *HSD17B13* and rs2642438A>G in *MTARC1*. Circulating levels of 1449 metabolites were measured using targeted and untargeted metabolomics. Associations between NASH-fibrosis variants and metabolites were assessed using linear regression. The specificity of variant-metabolite associations were compared to metabolite associations with ultrasound-defined steatosis, gene variants linked to liver fat (in *GCKR*, *PPP1R3B* and *LYPLAL1*) and gene variants linked to cirrhosis (in *HFE* and *SERPINA1*). Each NASH-fibrosis variant demonstrated a specific metabolite profile with little overlap (8/97 metabolites) comprising diverse aspects of lipid metabolism. Risk alleles in *PNPLA3* and *HSD17B13* were both associated with higher 3-methylglutarylcarnitine and three variants were associated with lower lysophosphatidylcholine C14:0. The risk allele in *MTARC1* was associated with higher levels of sphingomyelins. There was no overlap with metabolites that associated with *HFE* or *SERPINA1* variants. Our results suggest a link between the NASH-protective variant in *MTARC1* to the metabolism of sphingomyelins and identify distinct molecular patterns associated with each of the NASH-fibrosis variants under investigation.

## Introduction

Non-alcoholic steatohepatitis (NASH) is a common, multifactorial condition that may progress to cirrhosis, liver failure and hepatocellular carcinoma (1). NASH affects approximately 20% of individuals with non-alcoholic fatty liver disease (NAFLD), which is strongly associated with obesity and insulin resistance (2). Certain single nucleotide polymorphisms (SNPs) have been linked with disease progression through the development of NASH and fibrosis, apparently independent of insulin resistance (3).

A combination of exome-wide and genome-wide association studies (GWAS) have led to the identification of five loci (rs738409 in *PNPLA3* (4), rs58542926 near *TM6SF2* (5), rs72613567 in *HSD17B13* (6), rs641738 near *TMC4*-*MBOAT7* (7–9) and rs2642438 in *MTARC1* (10)) linked to NASH (11), fibrosis (12) and hepatocellular carcinoma (HCC) (13) in patients with NAFLD. Following *in vitro* and *in vivo* experiments, rs738409C>G (p.Ile148Met) in *PNPLA3* has been identified as a regulator of hepatic lipolysis (14,15). However, the potential pathophysiological consequence of the other variants is less understood or entirely unknown, with respect to the *MTARC1* p.Thr165Ala variant (10).

Metabolite association studies are an established technique for exploring the role of gene variants (16–18). Serum metabolite and plasma lipid profiling has been used to investigate some of these NASH-fibrosis gene variants and to identify differences between healthy subjects and those with NAFLD (for example, higher alanine in patients with NAFLD) (19,20). Metabolomics and lipidomics have also been used to differentiate non-alcoholic fatty liver (NAFL, or simple `steatosis') from NASH (for example, higher phosphatidylcholine C32:0 in patients with NASH) in a selected cohort of adults who underwent liver biopsy (21).

We hypothesized that variants associated with NASH-fibrosis would be associated with perturbation of metabolic pathways among adults from population-based cohort studies. To study this, we examined the serum metabolite and lipid profile of five variants associated with NASH-fibrosis in two population-based cohorts. We aimed to discover novel gene-metabolite associations that may be involved in the pathogenesis of NASH.

## Results

### Baseline participant characteristics

A total of 9135 participants from the Fenland cohort study were included, where 2301 (25%) had ultrasound evidence of hepatic fat accumulation (Supplementary Material, Table S2). Steatosis was associated with male sex, higher waist-to-hip ratio, lower high density lipoprotein (HDL) cholesterol and higher fasting insulin (Supplementary Material, Table S2).

**Figure 1:**
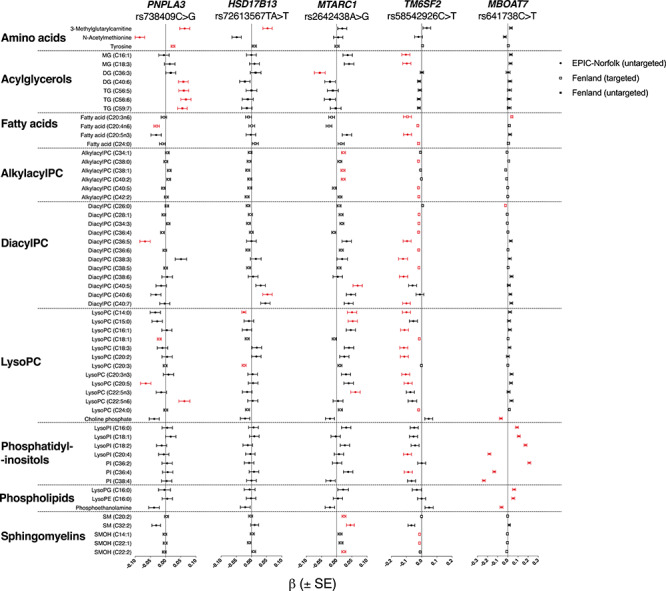
Associations of metabolites with NASH-fibrosis variants in the Fenland and EPIC-Norfolk cohort. Metabolites are arranged by class and annotated whether they were obtained by untargeted or targeted methods. Beta (β) ± standard error (SE) per allele from linear regression models are presented, adjusted for age, sex and the first ten genetic principal components and after standardization of each metabolite. Only metabolites significantly associated with at least one variant of interest (*Q* < 0.05) are presented and marked in Red. MG, monoglyceride; PC, phosphatidylcholine; PI, phosphatidylinositol; SM, sphingomyelin.

### Metabolite profile of hepatic steatosis

We first aimed to describe how hepatic steatosis may have influenced circulating metabolites. The serum metabolite profile of steatosis showed increased branched-chain amino acids and increased unsaturated short-chain triglyceride species, similar to the pattern observed for insulin resistance and dyslipidaemia. (Supplementary Material, Fig. S1 and Supplementary Material, Table S3). Adjusting for fasting insulin partially attenuated these changes (Supplementary Material, Fig. S1).

### NASH-fibrosis SNPs have specific metabolite profiles

We then proceeded to study the characteristics of individuals with risk alleles from the five NASH-fibrosis variants. None of the risk alleles for each SNP was associated with classical markers of insulin resistance (Supplementary Material, Tables S4–S8). For example, neither rs738409 C>G in *PNPLA3* nor rs58542926 C>T near *TM6SF2* (risk alleles for type 2 diabetes) (22) were significantly associated with differences in fasting insulin, glucose or HbA1c (Supplementary Material, Tables S4 and S5). Two variants did, however, associate with differences in lipoproteins that would classically be associated with a lower risk of atherosclerosis: lower LDL-cholesterol and total TG with rs58542926 C>T near *TM6SF2* (Supplementary Material, Table S5), and higher HDL-cholesterol with rs2642438 A>G in *MTARC1* (Supplementary Material, Table S8).

Next, we used targeted metabolite profiling (in 9135 participants) and untargeted lipidomics (in 1356 participants) from the Fenland cohort to test for SNP-metabolite associations. This analysis was complemented by a separate cohort of 9902 participants from the european prospective investigation into cancer and nutrition (EPIC)-Norfolk cohort (23) with untargeted metabolite profiling using a different platform for metabolomics measurements (Fig. 1). Each NASH-fibrosis SNP was found to have a specific metabolite profile (Table 1 and Supplementary Material, Table S3).

The strongest association for the rs738409 C>G in *PNPLA3* variant was observed with N-acetylmethionine (β = −0.09, *Q* = 5.7 × 10^−5^). The variant was further positively associated with plasma levels of TG and DG species carrying long-chain polyunsaturated fatty acids (Supplementary Material, Fig. S2), which was replicated in external datasets (Supplementary Material, Table S9). It was also positively associated with levels of 3-methylglutarylcarnitine (Fig. 1).

rs72613567 TA>T in *HSD17B13* was positively associated with long-chain diacylPC and negatively associated with short-chain lysoPC (Supplementary Material, Fig. S3 and Supplementary Material, Table S9). It was also positively associated with 3-methylglutarylcarnitine and pyroglutamine (Supplementary Material, Table S3).

rs58542926C>T near *TM6SF2* was inversely associated with plasma levels of several lipid classes, including diacylPC, alkylacylPC, fatty acids, lysoPC and sphingomyelins (Supplementary Material, Fig. S4). This was consistent across both cohorts and in external datasets (Supplementary Material, Tables S3 and S9). The magnitude of these associations was attenuated when adjusting for total cholesterol and triglycerides (Supplementary Fig. S5).

There was a trend towards lower TG and DG (in line with the main serum biochemistry results), though no individual species reached significance after adjusting for multiple testing (Supplementary Material, Fig. S4E and F). A similar trend in inverse associations with TG species was found in publicly available datasets (Supplementary Material, Fig. S6 and Supplementary Table S9).

The rs2642438 A>G risk variant in *MTARC1* was associated with higher sphingomyelins (Supplementary Material, Fig. S7), for example C20:2, (β = 0.02, *Q* = 0.015). This finding was replicated across several sphingomyelin species in both cohorts and in external datasets (Supplementary Material, Figs S3 and S6 and Supplementary Material, Table S9). This variant was also associated with higher diacylPC (18:0/22:5), higher lysoPC (C14:0 and C15:0) and higher alkylacyl PC (C34:1 and C40:2, for example, Supplementary Material, Fig. S7). The magnitude of these associations were minimally affected by adjusting for HDL cholesterol (Supplementary Material, Fig. S5).

Finally, rs641738 C>T near *MBOAT7* was found to have a strong association with phosphatidylinositols (PI), which was not replicated in any other NASH-fibrosis SNP (for example, PI C18:0/C20:4: β −0.2, *Q* = 1.7 × 10^–58^, Fig. 1). The variant was associated heterogeneously with species carrying omega-6 polyunsaturated fatty acids: for example, lower phosphatidylinositols (PI) with stearic acid and omega-6 polyunsaturated fatty acids (e.g. PI(18:0/20:4), β = −.24, *Q* = 1.7 × 10^−58^); higher PI with stearic acid and linoleic acid (e.g. PI(18:0/18:2), β = 0.21, *Q* = 1.1 × 10^−50^); and higher dihomo-gamma-linolenic acid (DHGL, Supplementary Material, Fig. S8). These findings were replicated in external datasets (Supplementary Material, Table S9).

**Table 1:** Top associations between metabolites and NASH-fibrosis variants in the EPIC-Norfolk and Fenland cohorts

Group	Metabolite	Beta (SE)	*P*	*Q*	*n*	Cohort	Method
rs72613567TA>T in *HSD17B13*							
Amino acid	3-methylglutarylcarnitine	.05 (.02)	5.3E-04	0.024	9756	EPIC	Untargeted
Phosphatidylcholines	LysoPC (C14:0)	−.02 (.01)	1.3E-04	0.006	9317	Fenland	Targeted
Phosphatidylcholines	LysoPC (C20:3)	−.02 (.01)	2.9E-04	0.012	9317	Fenland	Targeted
Phosphatidylcholines	DiacylPC (C40:6)	.05 (.02)	6.3E-04	0.028	9902	EPIC	Untargeted
rs641738C>T near *MBOAT7*							
Fatty acid	Fatty acid (C20:3n6)	.04 (.01)	2.5E-04	0.010	4279	Fenland	Targeted
Lysolipid	LysoPI (C20:4)	−.18 (.01)	9.3E-38	3.0E-34	9902	EPIC	Untargeted
Lysolipid	LysoPI (C18:2)	.17 (.01)	2.1E-34	5.2E-31	9902	EPIC	Untargeted
Phosphatidylcholines	DiacylPC (C26:0)	−.02 (.01)	0.001	0.037	9317	Fenland	Targeted
Phosphatidylinositol	PI (C38:4)	−.24 (.01)	1.8E-62	1.7E-58	9902	EPIC	Untargeted
Phosphatidylinositol	PI (C36:2)	.21 (.01)	1.1E-50	5.4E-47	9902	EPIC	Untargeted
rs2642438A>G in *MTARC1*							
Lysolipid	LysoPC (C22:5)	.06 (.02)	6.5E-05	0.005	9858	EPIC	Untargeted
Lysolipid	LysoPC (C15:0)	.05 (.02)	6.1E-04	0.028	9901	EPIC	Untargeted
Lysolipid	LysoPC (C14:0)	.05 (.02)	0.001	0.039	9901	EPIC	Untargeted
Phosphatidylcholines	DiacylPC (C40:5)	.07 (.02)	7.5E-06	8.6E-04	9901	EPIC	Untargeted
Phosphatidylcholines	Alkylacyl PC (C34:1)	.02 (.01)	8.6E-04	0.026	9317	Fenland	Targeted
Sphingomyelin	SM (C20:2)	.02 (.01)	3.9E-04	0.015	9317	Fenland	Targeted
Sphingomyelin	SMOH (C22:2)	.02 (.01)	5.4E-04	0.019	9317	Fenland	Targeted
Sphingomyelin	SM (C32:2)	.05 (.01)	3.9E-04	0.019	9901	EPIC	Untargeted
rs738409C>G in *PNPLA3*							
Amino acid	N-acetylmethionine	−.09 (.02)	3.6E-07	5.8E-05	9893	EPIC	Untargeted
Amino acid	Tyrosine	.02 (.01)	1.3E-04	0.006	9317	Fenland	Targeted
Amino acid	3-methylglutarylcarnitine	.06 (.02)	1.2E-04	0.008	9756	EPIC	Untargeted
Fatty acid	Fatty acid (C20:4n6)	−.03 (.01)	0.001	0.037	4279	Fenland	Targeted
Triacylglyceride	TG (C56:6)	.07 (.02)	7.3E-05	0.004	1356	Fenland	Untargeted
Triacylglyceride	TG (C56:5)	.06 (.02)	2.6E-04	0.011	1356	Fenland	Untargeted
Triacylglyceride	TG (C59:7)	.06 (.02)	5.6E-04	0.019	1356	Fenland	Untargeted
rs58542926C>T in *TM6SF2*							
Fatty acid	Fatty acid (C20:4n6)	−.03 (.01)	1.9E-05	0.001	4279	Fenland	Targeted
Fatty acid	Fatty acid (C20:3n6)	−.09 (.03)	3.7E-04	0.019	9891	EPIC	Untargeted
Lysolipid	LysoPC (C14:0)	−.1 (.03)	1.7E-04	0.010	9901	EPIC	Untargeted
Phosphatidylcholines	DiacylPC (C34:4)	−.03 (.004)	2.0E-10	7.8E-08	9317	Fenland	Targeted
Phosphatidylcholines	DiacylPC (C34:4)	−.15 (.03)	1.0E-08	2.5E-06	9901	EPIC	Untargeted
Phosphatidylcholines	Alkylacyl PC (C40:5)	−.02 (.004)	1.0E-07	2.3E-05	9317	Fenland	Targeted
Phosphatidylcholines	DiacylPC (C34:4)	−.15 (.03)	1.7E-07	3.0E-05	7991	EPIC	Untargeted
Phosphatidylcholines	Alkylacyl PC (C38:0)	−.02 (.004)	1.8E-07	3.2E-05	9317	Fenland	Targeted
Sphingomyelin	SMOH (C22:1)	−.01 (.004)	7.3E-04	0.023	9317	Fenland	Targeted
Sphingomyelin	SMOH (C14:1)	−.01 (.004)	0.001	0.038	9317	Fenland	Targeted

Beta represents the change in 1 normalized standard deviation of each metabolite per effect allele (Supplementary Material, Table S8 for a full list of significant associations, *Q* < 0.05). DG, diacylglycerol; GPC, glycerophosphatidylcholine; GPI, glycerophosphatidylinositol; PC, phosphatidylcholine; *Q*, *Q*-value (false discovery rate-corrected *P*-value); SM, sphingomyelin; SMOH, sphingomyelin hydroxide; TG, triacylglycerol.

### Overlap between metabolite profiles

We compared SNP-metabolite associations to assess whether there were clear trends that reflected steatosis, NASH, or fibrosis. Out of the 97 different metabolites significantly associated with at least one of the five NASH-fibrosis variants, only 8.2% (8/97) were associated with two or more variants (Supplementary Material, Table S3). For all eight metabolites, the directions of the associations were consistent across SNPs effect (Fig. 1). For example, risk alleles near or in *TM6SF2, HSD17B13* and MTARC1 were all associated with lower plasma levels of lysoPC (C14:0, Fig. 1). Similarly, higher levels of 3-methylglutarylcarnitine were associated with risk alleles in *PNPLA3* and *HSD17B13* (Fig. 1).

Next, we assessed which of the five variants contributed most to the overall variation in these metabolites. In the Fenland cohort, rs738409 C>G in *PNPLA3* accounted for the most variability (adjusted R^2^ 5.8%) in the 41 significantly associated metabolites. Compared to 3.4% for rs72613567 TA>T in *HSD17B13*, 3.2% for rs58542926C>T near *TM6SF2*, 1.1% for rs641738 C>T near *MBOAT7* and 0.0% for rs2642438 A>G in *MTARC1*.

### Comparison with *HFE* and *SERPINA1*

To determine whether these results were specific to NASH-fibrosis or were more generally reflective of hepatocyte dysfunction, we compared associations from the five NASH-fibrosis variants with SNP-metabolite associations for *HFE* and *SERPINA1* variants. A total 37 metabolites were associated with rs1800562 G>A in *HFE* (p.Cys282Tyr) or rs28929474 C>T in *SERPINA1* (p.Glu366Lys), variants linked to metabolic cirrhosis (Supplementary Material, Table S3). There was no overlap between these 37 metabolites and the 97 associated with NASH-fibrosis variants (Supplementary Material, Table S3).

### Comparison with steatosis and insulin resistance

Finally, we aimed to test whether the SNP-metabolite associations were indicative of steatosis or insulin resistance or both. In the Fenland cohort, out of 41 different metabolites associated with the five different NASH-fibrosis variants, 78% (32/41) were also significantly altered in steatosis (Supplementary Material, Table S3). The majority (69%, 22/32) were PC species though the direction of change was not consistent (Supplementary Material, Table S3). For example, several NASH-fibrosis variants were associated with lower levels of lysoPC (C14:0), whereas steatosis was associated with higher levels (β = 0.07, *Q* = 7.7x10^−8^, Supplementary Material, Fig. S1 and Supplementary Material, Table S3).

Liver-fat related variants in *GCKR*, *PPP1R3B* and *LYPLAL1* were associated with the plasma levels of 281 metabolites. Fifty-two per cent (50/97) of metabolites associated with NASH-fibrosis variants were also associated with liver fat variants, again the majority (86%, 43/50) were PC species due to similarity between *GCKR* and *TM6SF2* metabolite associations (Supplementary Material, Table S3).

In order to determine whether these SNP-metabolite associations were indicative of insulin resistance or body composition, we compared the metabolite associations of published genetic risk scores with those from NASH-fibrosis SNPs. There was some overlap between metabolites associated with NASH-fibrosis variants and genetic risk scores for BMI, BMI-adjusted waist-to-hip ratio, body fat percentage and insulin resistance. Out of 97, 24 different metabolites (24.7%) were also associated with one of the four genetic risk scores (Supplementary Material, Table S3), most of which (54%, 13/24) were PC species, pointing to shared pathways between higher genetic susceptibility to adverse body composition or insulin resistance and NASH.

## Discussion

There is strong human genetic evidence for five common variants in the pathogenesis of fibrotic NASH. To improve our understanding about functional consequences of these SNPs, we used blood metabolite profiling in two large population cohorts. Metabolite profiles were found to be highly specific with only a few metabolites significantly associated with more than one variant, such as 3-methylglutarylcarntine (*PNPLA3* and *HSD17B13*) and lysoPC C14:0 (*TM6SF2, HSD17B13* and *MTARC1*) that were not replicated in other variants associated with liver fat but not fibrosis (e.g. *LYPLAL1*) or fibrosis without steatosis (e.g. *SERPINA1*). The specific (lipid) profiles of each variant might be interpreted as distinct hits on lipid metabolism resulting in the same consequence, accelerating progression from NAFLD to NASH.

rs2642438 A>G in *MTARC1* (p.Thr165Ala) has very recently been identified as a risk variant in NAFLD and all-cause cirrhosis (10). *MTARC1* encodes for an outer mitochondrial membrane-bound molybdenum enzyme recognized to be involved in drug metabolism and has the capacity for nitrite reduction. Its function in liver disease is largely unknown (24–27). We found that this *MTARC1* variant was associated with higher levels of a range of sphingomyelin species across all studied cohorts and our results are supported by previous studies (28,29). Sphingomyelins are primarily plasma membrane components that are synthesized from ceramides. Higher sphingomyelins have been implicated in the development of cirrhosis (30,31) as well as insulin resistance (32,33). Several members of the ceramide-sphingomyelin pathway have been linked to lipotoxicity and it is likely that perturbation of the balance in this metabolic pathway is harmful in liver disease from multiple aetiologies (34). How this variant relates to the sphingomyelin pathway is unclear and requires further investigation, particularly given that there are many variants that affect sphingomyelin levels and have no identified impact on NAFLD, for example, variants in sphingosine-1-phosphate phosphatase 1 (*SGPP1*) (35).

Along with rs58542926 near *TM6SF2,* rs2642438 A>G in *MTARC1* was found to be associated with changes in major classes of serum lipoparticles. Metabolite associations for rs2642438 A>G remained after adjusting for HDL cholesterol levels, however further refined studies (such as lipidomics separated by lipoparticles) are needed to give biological insight into these observations. Mechanistic studies, similar to those performed for TM6SF2 (36), would help to identify the role MTARC1 plays in lipoparticle export and/or lipidation.

rs738409 C>G (p.Ile148Met) in *PNPLA3* is the genetic variant most strongly associated with hepatic outcomes in NAFLD. Three recent studies have provided compelling evidence that *PNPLA3* binds to ABHD5 (abhydrolase domain containing 5) and regulates adipose triglyceride lipase (ATGL)-mediated hydrolysis from lipid droplets (14,15,37). We found this variant to be associated with higher TG with long-chain polyunsaturated fatty acids, consistent with mouse data and other human studies (38–40). These have been classed as ‘healthy lipids’ (41) and are not associated with insulin resistance (42,43), despite this variant being positively associated with T2DM (22). These data suggest that the length and saturation of TG fatty acids may influence or depend on the *PNPLA3*-ABHD5-ATGL interaction.

The TA-duplication allele of rs72613567 in *HSD17B13* reduces the risk of NAFLD-cirrhosis and mitigates the risk conferred by carriage of the G-allele in *PNPLA3* (44). Abul-Husn *et al*. found that the encoded enzyme 17β-Hydroxysteroid dehydrogenase type 13 to have activity on several steroids and bioactive lipids and did not influence total hepatocyte TG *in vitro*, however, a mouse knockout did have increased hepatic steatosis (45). It has been recently reported to act as a hepatic retinol dehydrogenase (46), but we did not find an effect on plasma retinol levels. We also found lower circulating lysoPC species, which is consistent with the recent findings of Luukkonen *et al.* (47) who analysed the hepatic lipid profile of NAFLD patients carrying the protective variant (rs72613567T>TA) and identified higher hepatic phospholipids.

One metabolite of interest is 3-methylglutarylcarnitine, where lower levels were associated with *HSD17B13* (protective) and higher levels were associated with *PNPLA3* (harmful). This metabolite is typically elevated in 3-hydroxy-3-methylglutaryl-coenzyme A lyase deficiency (48), a Reye syndrome-like inborn error of metabolism that presents with microvesicular steatosis and liver failure. This enzyme is also necessary for metabolizing β-hydroxy β-methylglutaryl-coenzyme A towards ketogenesis and away from cholesterol synthesis (49). The consistent direction of association across two NASH-fibrosis variants with this pathway warrants further investigation. However, it should be noted that we were unable to validate this finding in any external cohort, therefore further replication is also needed.

rs641738C>T near *TMC4*-*MBOAT7* was initially identified at genome-wide significance for alcoholic liver disease but a recent meta-analysis has demonstrated it to be associated with NASH, fibrosis and HCC (9). Its function remains unclear as the variant lies within *TMC4*, a putative transmembrane transporter with no known role in the liver. It also lies close to *MBOAT7*, an acyltransferase that incorporates (very) long-chain polyunsaturated fatty acids into lysophosphatidylinositols (7,29,50–52) and rs641738C>T is reported to be associated with reduced liver expression of *MBOAT7* (7,53). We found this variant to be very strongly associated with greater DHGL or higher or lower phosphatidylinositols with different omega-6 fatty acids. These data suggest that rs641738C>T near *TMC4*-*MBOAT7* results in reduced *MBOAT7* activity, which leads to differential degrees of acylation of different omega-6 polyunsaturated fatty acids. Therefore, these metabolomics data provide evidence for *MBOAT7* as the causal gene in this locus. Its underlying mechanism and clinical significance are unclear, but still implicative of its relevance to type 2 diabetes epidemics, in addition to NASH, because we previously found heterogeneous associations of omega-6 fatty acids with type 2 diabetes incidence (54). Further mechanistic work is warranted to understand how this variant influences the development of NASH.

We found metabolite changes to be relatively specific to each variant, rather than common and reflect NASH or fibrogenesis. There was only an 8% overlap in metabolites between the five NASH-fibrosis metabolites though where overlap did occur, the direction of association was consistent between SNP-metabolite pairs. No metabolite associations were shared with *HFE* or *SERPINA1* variants, which suggests levels of serum metabolites reflect specific metabolic pathway perturbations, rather than a generic response to hepatocyte dysfunction. PC species were the most ubiquitously altered metabolites and accounted for the majority of overlap between steatosis and NASH-fibrosis variants. One might speculate that PC species are surrogate markers of lipoprotein metabolism reflecting the final effect of impaired liver function in the circulation due to intrahepatic deteriorations in lipid metabolism accumulating during lifetime in risk allele carriers. Other studies have shown that NAFLD is associated with non-specific changes in amino acids and phospholipids, which have also been linked to insulin resistance (55–63). These findings suggest common genetic variants influence the perturbation of different metabolic pathways, which may result in NASH being a metabolically heterogeneous disease.

There are four broad processes that can affect liver fat: *de novo* lipogenesis, import of lipids, export of lipids and metabolic breakdown of lipids. It is likely that each of these plays some role in the development and progression of NAFLD. Each will also contribute to the serum metabolite profile in different ways and this may be reflected in our results. From this perspective, it may not be surprising that there is minimal overlap in the serum metabolite profile associated with each variant.

Two studied variants (rs738409C>G in *PNPLA3* and rs58542926 near *TM6SF2*) are positively associated with type 2 diabetes through large GWAS (22) yet we found them not to be associated with fasting insulin or glucose. Detailed studies (including the use of hyperinsulinaemic clamps) in smaller groups of individuals have yielded similar results (40,64,65). This observation could be explained if the development of hepatic steatosis in response to these variants was the intermediate causal step. Both of these variants are strongly associated with higher liver fat and steatosis itself may affect systemic insulin resistance (66,67). Experimentally dissecting these mechanisms is challenging as it would require sensitive measurements in individuals matched for adiposity and liver fat, yet stratified by genotype with sufficient power to demonstrate effects.

The main strength of this study is that it is based on two well-characterized, population-based cohorts. Our study evaluated 19 037 in total whereas previous relevant work has analysed data from up to 1810 adults from population-based cohorts (20) or 695 biopsied NAFLD patients (68). For many lipid classes, we were able to validate our results across multiple metabolomics platforms and independent cohorts, thought difference in the platforms may mean that the beta-regression coefficients are not directly comparable. This is also the first study to report characterization of the *MTARC1* variant in a population-based cohort. In addition, our conclusions should be free from potential selection bias of previous NAFLD or NASH cohorts or case–control studies (21,68,77–81,69–76).

A potential limitation of our study is the use of ultrasound for definition of steatosis, rather than quantitative magnetic resonance spectroscopy or proton density fat fraction. Ultrasound identification of hepatic steatosis was not available in the EPIC-Norfolk cohort. Furthermore, we did not have a complete overlap of metabolomics between Fenland and EPIC-Norfolk cohorts, which reduced the power for identifying associations in those analyses. This meant that we were unable to explore some specific steatosis-metabolite associations, for example phosphatidylinositols, which were only covered by untargeted profiling in the EPIC-Norfolk cohort. In addition, we did not have detailed data on liver-related outcomes (for example, progression of fibrosis or development of NASH) or aspartate aminotransferase, therefore were unable to calculate non-invasive scores of hepatic fibrosis. Because of our cross-sectional design, we could not determine the causality whether metabolites affected hepatic steatosis or vice versa, whereas gene-metabolite associations should be free from the concern. As population-based cohorts comprised of healthy individuals, this study did not have liver biopsy data. Therefore, we were unable to correlate metabolite changes to histological stage of disease or assess for effect on the hepatic metabolite profile. These would be important future studies to validate these findings.

It should be noted that other variants in (or near) the five studied genes show strong variant-metabolite associations, though have not been associated with liver disease to date. For example, Draisma *et al.* (28) found rs2576452C>T near *MBOAT7* was negatively associated with 1-arachidonoylglycerophosphoinositol, *P* = 8.8 × 10^−18^. We selected these five variants for inclusion based on their clear and replicated associations with NASH-fibrosis however studying other variant-metabolite associations in *PNPLA3*, *TM6SF2*, *MBOAT7*, *MTARC1* and *HSD17B13* may reveal additional insights into the function of these genes.

There are some limitations of the techniques used for metabolite profiling in this study. There is a risk of misidentification of species from untargeted metabolomics, which is most relevant for some metabolites where we were unable to validate trends using targeted metabolomics from the Fenland (or an external) cohort, for example 3-methylglutarylcarnitine. In addition, the platform used with the EPIC-Norfolk cohort has a preference towards hydrophilic metabolites and may be less accurate with more hydrophobic metabolites. Data on triglycerides and diglycerides were only available from the Fenland cohort and in a small number of individuals (1356), which limited power for detecting associations and performing replication, particularly for *TM6SF2*. Though reassuringly, our results for rs738409C>G in *PNPLA3* are consistent with previous reports (40,64,82).

In conclusion, metabolite profiling from two large cohort studies demonstrates a specific signature of pathway perturbation associated with five NASH-fibrosis variants. For example, *MTARC1* p.Thr165Ala is associated with higher sphingomyelin species. These findings suggest that common genetic variants may influence different pathophysiologic pathways in the development of NAFLD.

## Materials and Methods

### Fenland study cohort

The Fenland study is a population-based cohort of 12 435 individuals recruited in 2005–2015 from general practice lists from Cambridgeshire, UK. The study and its methods were described in detail previously (83). Briefly, the study aimed to examine genetic, metabolic, lifestyle and societal determinants for the development of type 2 diabetes and related metabolic disorders. Therefore, all the participants were eligible if they were free from type 2 diabetes prior to the first study visit. Participants underwent detailed metabolic phenotyping, genome-wide genotyping and serum metabolomic profiling. For inclusion in the current analysis, participants must have had an abdominal ultrasound scan (US) for determination of hepatic steatosis, densely imputed genotype data for the SNPs of interest (as described below) and body composition analysed by either dual-energy X-ray absorptiometry (DXA) or bioelectrical impedance analysis (BIA). Furthermore, participants were excluded if: no genotype data were available, related individuals and genetic ancestry outliers. Applying those criteria left 9135 participants to be included in statistical analyses. The study was approved by the Cambridge Local Research Ethics Committee (ref: 04/Q0108/19) and all participants provided written informed consent to participate in the study. Sociodemographic factors (e.g. ethnicity, sex) and alcohol use were extracted from the Fenland General Questionnaire.

### Measures of body composition—Fenland cohort

Anthropometric measures and DXA were described in detail elsewhere (83). In brief, participants’ height, weight, waist- and hip- circumferences were measured. Body mass index (BMI) was calculated by weight (kg) divided by squared height (m^2^). DXA (Lunar Prodigy Advanced fan beam scanner, GE Healthcare, Hatfield, UK) estimated lean and fat mass with their relative distributions using the GE software (version no. 14; GE Healthcare). BIA was performed using TANITA bc-418 MA body fat monitor (Tanita, Tokyo, Japan) to estimate total body fat percentage. Body fat percentage was used from DXA where available and from BIA if unavailable. All measurements were performed by trained operators.

### Abdominal US—Fenland cohort

Liver US images were taken for the Fenland cohort, as described elsewhere (84). The images were recorded and scored retrospectively by two operators who were blinded to all other study measures. The ultrasound images were acquired using the LOGIQ Book and Logic GE Healthcare ultrasound systems with 3C MHz-RS and 2–5 MHz 3C-RC curved array transducers, respectively, and were qualitatively scored according to standardized criteria (85–88). The hepatic steatosis scoring criteria were: Criterion 1, increased echo reflectivity of the liver parenchyma (bright liver in comparison with the kidney); Criterion 2, decreased visualization of the intrahepatic vasculature; Criterion 3, attenuation of ultrasound beam. Each criterion was scored on a 4-point scale (i.e. as 1, 2, 3 or 4) and summed, resulting in cumulative liver fat score (range: 3 to 12). A score of ≤ 4 was classified as normal liver and ≥ 5 was classified as steatosis.

### EPIC-Norfolk study cohort

The EPIC-Norfolk study is a prospective population cohort of over 25 000 individuals aged 40–79 years at recruitment living in Norfolk, UK (baseline years = 1993–1997) (89), nested within the European Prospective Investigation into Cancer and Nutrition (EPIC). The study was approved by the Norfolk Research Ethics Committee (ref. 05/Q0101/191) and all participants gave their written consent on entering the study. Metabolomics, genotyping and clinical outcome data were available for 9902 participants. No liver imaging (for identification of steatosis) was available in this cohort.

### Genotyping data—Fenland and EPIC-Norfolk Cohorts

Participants were genotyped using Affymetrix Axiom UKBiobank, Affymetrix 500 K Array Set and Illumina Infinium Core Exome 24v1 arrays. Results were imputed to the HRC and UK10k panels, followed by a combination of imputation results.

Genotype was extracted for five SNPs associated with NASH-cirrhosis: rs738409C>G in *PNPLA3* (NC_000022.11:g.43928847C>G/NP_079501.2:p.Ile148Met), rs58542926C>T near *TM6SF2* (NC_000019.10:g.19268740C>T/NP_001001524.2:p.Glu167Lys), rs641738C>T near *TMC4*-*MBOAT7* (NC_000019.10:g.54173068T>C/NP_001138775.2:p.Glu17Gly, referred to as ‘*MBOAT7*’), rs72613567TA>T in *HSD17B13* (NC_000004.12:g.87310241dup), and rs2642438A>G in *MTARC1 (*NC_000001.11:g.220796686A>G/NP_073583.3:p.Thr165Ala). Call rate was >98% for all variants.

In addition, genotype was extracted for three variants at genome-wide significance for liver fat [rs780094C>T in *GCKR* (NC_000002.12:g.27518370C>T), rs4240624G>A near *PPP1R3B* (NC_000008.11:g.9326721G>A), rs12137855T>C in *LYPLAL1* (NC_000001.11:g.219275036C>T)] and variants associated with haemochromatosis (rs1800562G>A in *HFE* [NC_000006.12:g.26092913G>A/NP_000401.1:p.Cys282Tyr)] and alpha-1-antitrypsin deficiency (rs28929474C>T in *SERPINA1* (NC_000014.9:g.94378610C>G/NP_001002236.1:p.Glu366Lys). These three variants in or near *GCKR*, *PPP1R3B* and *LYPLAL1* are well-established determinants of liver fat but do not appear to associated with cirrhosis, therefore were included for comparison against the NASH-fibrosis variants (10,90) as ‘steatosis only’ variants. Whilst the variants in *HFE* and *SERPINA1* are associated with cirrhosis but not liver fat by reasonably well-established metabolic pathways. These were included for comparison against NASH-fibrosis variants to assess whether metabolite associations were variant-specific or whether they non-specifically reflected hepatic fibrosis. A summary of the evidence for the inclusion of variants in the analysis is presented in Supplementary Material, Table S1. Finally, genotyping data were used to calculate previously published SNP scores for metabolic traits: BMI (91), BMI-adjusted waist-to-hip ratio (92), body fat percentage (93) and insulin resistance (94).

### Metabolomics profiling and gas chromatography of fatty acids—Fenland cohort

Fasting serum was used for targeted metabolomics using AbsoluteIDQ p180 kit (BIOCRATES Life Sciences AG, Innsbruck, Austria), which includes: 24 amino acids, 10 amines, 40 carinitines, 14 lysophosphatidylcholines (lyso-PC), 37 diacylphosphatidylcholines (diacyl-PC), 37 alkylacylphosphatidylcholines (alkylacyl-PC), 11 sphingomyelins (SM) and the sum of hexoses (95). The panel was measured using ABSciex 5500 Qtrap with a Waters Acquity UPLC as described elsewhere (63,95). In addition, 37 fatty acids were measured using an automated, high-throughput gas chromatography method in a subset (*n* = 4266), as described previously (96). In short, the plasma phospholipid fraction was obtained using solid phase extraction and hydrolysed. Isolated fatty acids were then methylated, yielding fatty acid methyl esters (FAME) and separated by gas chromatography (J&W HP-88, 30 m length) equipped with flame ionization detection (7890N GC Agilent Technologies, USA). Samples were processed in a random order, and laboratory staff was blinded to any participant characteristics. Fatty acids were identified by their retention times compared with those of commercial standards and expressed as a per cent of total phospholipid fatty acids (mol%). All assays were performed according to the manufacturers’ instructions.

### Lipidomics—Fenland cohort

Untargeted lipidomic measurement was performed on fasting serum for a subset of participants (*n* = 1356), as previously described (77). Briefly, samples were diluted with 100 μl of MilliQ H_2_O in a well of a glass-coated 2.4 ml deep well plate (Plate+TM, Esslab, Hadleigh, UK), then 250 μl of MeOH was added. Lipids were partitioned into 500 μl of Methyl-tertiary-butyl ether. After centrifugation, the organic layer was concentrated and used for lipid analysis. Samples were infused into a Thermo Exactive benchtop orbitrap (Hemel Hampstead, UK), using an Advion Triversa Nanomate (Ithaca, USA) and data acquired in both positive (+1.2 kV) and negative (−1.5 kV) mode voltages. All experiments were run with blank controls and two different quality control samples. In total, 218 lipid signals were detected and annotated as described previously (77) based on the identification at level 2 of the Metabolomics Standards Initiative. Identified lipid species included: 8 alkenylphosphatidylcholines (alkenyl-PC), 11 alkylacylphosphatidylcholines (alkylacyl-PC), 10 cholesterol esters (ce), 32 diacylphosphatidylcholines (diacyl-PC), 9 lysophosphatidylcholines (lyso-PC), 10 phosphoethanolamines (PE), 19 sphingomyelins (SM), 32 diacylglycerols (DG) and 41 triacylglycerols (TG).

### Untargeted metabolomics—EPIC-Norfolk cohort

Untargeted metabolomics was measured using the DiscoveryHD4® platform (97) (Metabolon, Inc., Durham, USA), which uses a Hydrophilic Interaction Liquid Chromatographic method in non-fasted citrated plasma samples, in two quasi-randomly selected substudies. Metabolite levels were median-normalized across rundays and no imputation of missing values was performed. All the analyses were performed for each dataset separately. The reported analyses included 9902 individuals with full covariate information and plasma levels of 977 metabolites. Fixed-effects meta-analysis was used to combine the metabolite results from the two datasets.

## Statistics

In both cohorts, anthropometry, body composition measurements, metabolomics and lipidomics variables were transformed logarithmically. Metabolite levels were further winsorised (to 5 sd) and standardized (μ = 0, sd = 1) using statistics specific to each dataset.

In the Fenland study cohort, characteristics of participants with and without hepatic steatosis were compared using linear regression for continuous variables and logistic regression for categorical variables, adjusting for age, sex and BMI. NAFLD-associated genotypes were assessed for associations with anthropometric and metabolic traits using linear regression (coding effect allele dosage as 0, 1 and 2) adjusting for age, sex and the first ten principal components from a principal component analyses on the genetic data to account for population stratification, as has been used previously by our group (63,98,99).

Targeted metabolomic and untargeted lipidomic profiles were compared between participants with and without hepatic steatosis. The semi-quantitative score of the degree of steatosis was analysed as an ordinal variable in secondary analysis. Associations between metabolites and steatosis were first assessed using logistic regression corrected for age, sex and population stratification. Each metabolite was used as a dependent variable and either steatosis status (yes/no) or steatosis score (from 3 to 12 points) was used as the independent variables. To illustrate the effect of insulin resistance on these associations, the analysis was repeated additionally correcting for BMI and fasting insulin.

Associations of metabolite levels with SNP effect alleles were assessed using linear regression adjusted for age, sex and population stratification, for all SNPs. Each metabolite was used as a dependent variable and number of risk alleles (0, 1 or 2) was used as the independent variable.

Variants in rs58542926C>T near *TM6SF2* and rs2642438A>G in *MTARC1* were found to influence total serum lipids and lipoprotein levels. Therefore, regression analyses were repeated for these variants additionally adjusting for: total cholesterol and total triglycerides for rs58542926C>T near *TM6SF2*; and, HDL cholesterol for rs2642438A>G in *MTARC1*.

To provide further confidence in the validity of our results, we tested metabolite-variant associations in random subgroups of the cohorts. The Fenland cohort was randomly divided into two groups (g1 & g2) and regression analyses were repeated. The EPIC-Norfolk cohort was already comprised of two substudies (g1 & g2). Metabolite-variant associations were considered significant where: overall *Q*-value < 0.05 and there was a directionally consistent beta-regression coefficient across g1, g2 and the overall cohort. Next, we examined classes of metabolites [e.g. triglycerides, diacylphosphatidylcholines (diacylPC)] for consistent trends in variant-metabolite associations across different analysis platforms and cohorts. Beta-regression coefficients were plotted against the number of carbons and/or double-bounds in lipid chains. Simple linear regression was performed on these plots, as a form of meta-regression, separately for each cohort (and metabolomics platform).

For external validation of our results, we compared our results against publicly available datasets, where possible (17,28,29,100–103). These were examined for directionally consistent associations across classes of metabolites.

In order to determine which of the five NASH-fibrosis variants was most informative in the variation of relevant metabolites, we performed multiple linear regression in the Fenland cohort. Each of the 41 metabolites associated with at least one of the variants were included as dependent variables and number of risk alleles (0, 1 or 2) was used as the independent variable. Adjusted R^2^ was recorded as a measure of relative contribution to the variation in the included metabolites.

Benjamini–Hochberg correction for multiple testing was used throughout with *Q*-value < 0.05 considered significant.

Statistical analyses were conducted using Stata v14.1 (StataCorp), GraphPad Prism (v8.0 for Mac, GraphPad Software, La Jolla California, USA) and R 3.5.1.

## Supplementary Material

Mann_HMG-2020-TF-00173_Supplement_clean_v2_ddaa162

Mann_HMG-2020-TF-00173_Supplement_clean_ddaa162

Mann_HMG-2020-TF-00173_Supplement_clean_v2_ddaa162

Mann_HMG-2020-TF-00173_SupTables3-9_ddaa162
