# Supplementary material for: Insights into genetic variants associated with NASH-fibrosis from metabolite profiling
Source: Hum Mol Genet. 2020 Jul 28;29(20):3451–63. doi: 10.1093/hmg/ddaa162 (PMC7116726; doi:10.1093/hmg/ddaa162)
Supplement: Mann_HMG-2020-TF-00173_Supplement_clean_v2_ddaa162 [file mann_hmg-2020-tf-00173_supplement_clean_v2_ddaa162.pdf]

Insights into genetic variants associated with NASH-fibrosis from metabolite profiling

Supplementary Data

SUPPLEMENTARY FIGURES 2

SUPPLEMENTARY FIGURE 1. 2
SUPPLEMENTARY FIGURE 2. 3
SUPPLEMENTARY FIGURE 3. 4
SUPPLEMENTARY FIGURE 4. 5
SUPPLEMENTARY FIGURE 5. 7
SUPPLEMENTARY FIGURE 6. 9
SUPPLEMENTARY FIGURE 7. 8
SUPPLEMENTARY FIGURE 8. 10

SUPPLEMENTARY TABLES 12

SUPPLEMENTARY TABLE 1.......... 12
SUPPLEMENTARY TABLE 2. 13
SUPPLEMENTARY TABLE 3 [EXCEL SPREADSHEET]. 13
SUPPLEMENTARY TABLE 4. 14
SUPPLEMENTARY TABLE 5. 15
SUPPLEMENTARY TABLE 6. 16
SUPPLEMENTARY TABLE 7. 17
SUPPLEMENTARY TABLE 8. 18
SUPPLEMENTARY TABLE 9 [EXCEL SPREADSHEET]. 18

SUPPLEMENTARY REFERENCES 19

Supplementary Figures

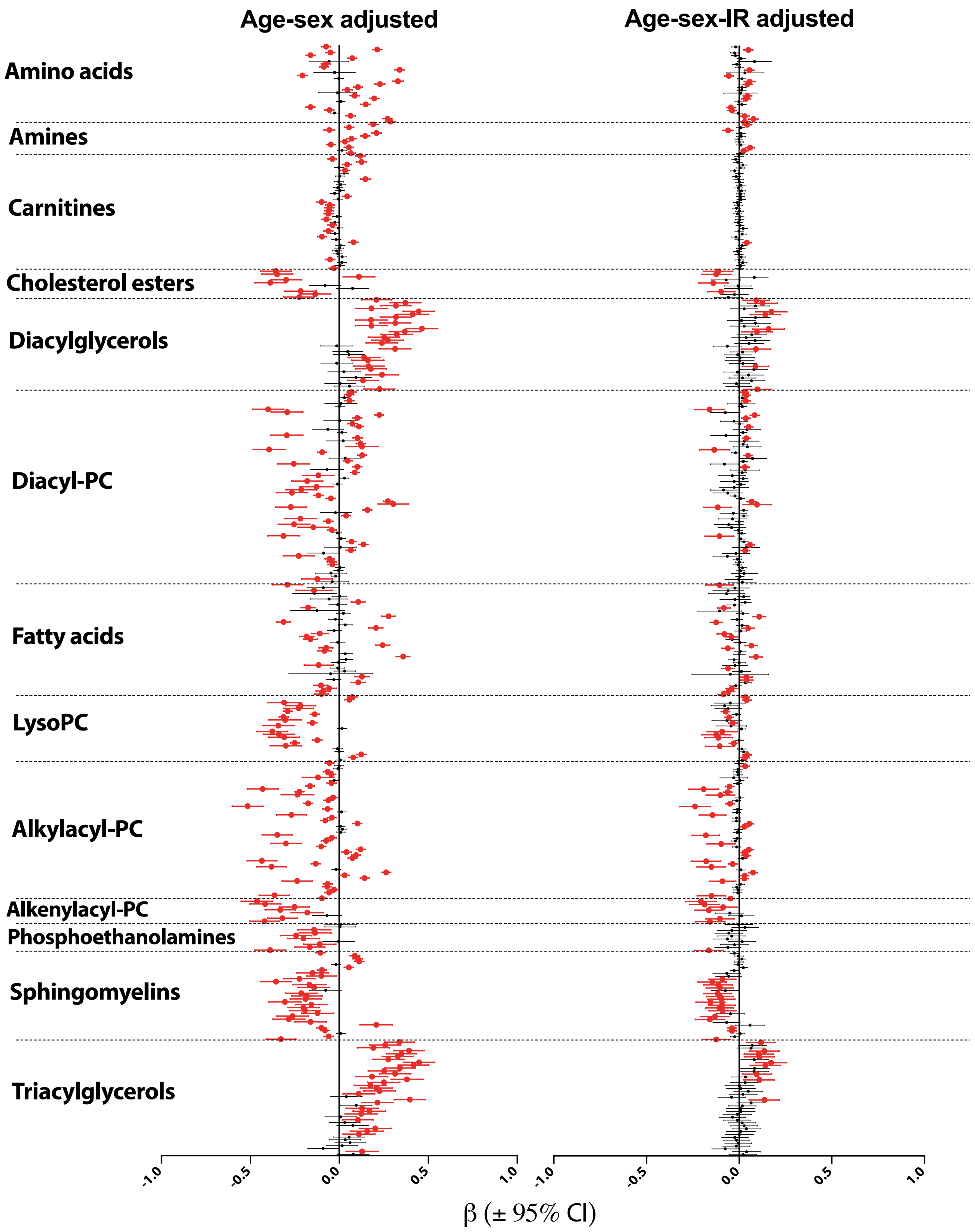

Supplementary Figure 1.
Association of metabolites with hepatic steatosis by logistic regression adjusted for age, sex, & BMI only [left] and age, sex, BMI, type 2 diabetes, and fasting insulin [right]. β (± 95% CI) represents the change in 1 normalised standard deviation of each metabolite for the presence of hepatic steatosis. Metabolites in red are associated with Q<0.05. IR, insulin resistance; PC, phosphatidylcholine.

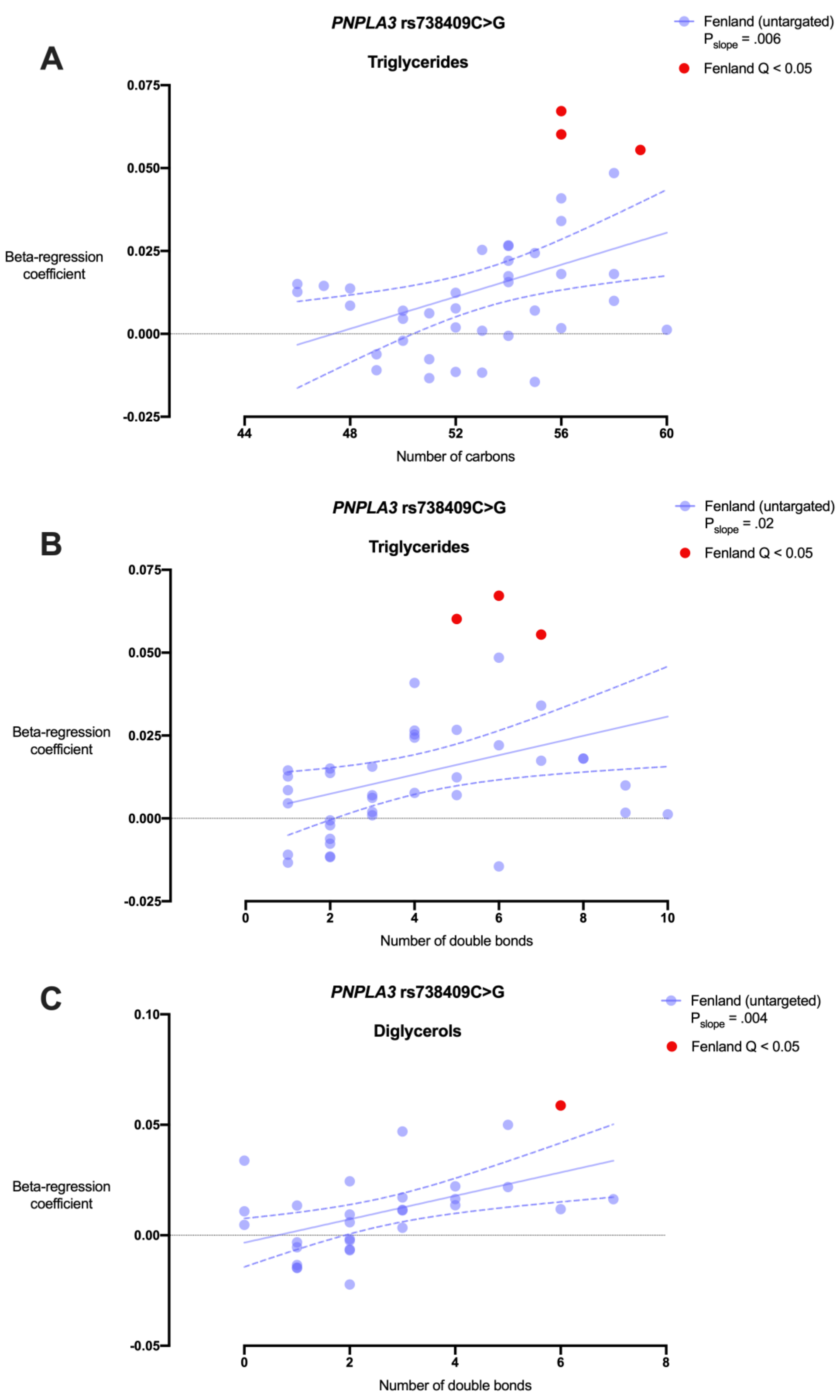

Supplementary Figure 2.

Characteristics of plasma lipid species associated with rs738409C>G in *PNPLA3*. The variant was more positively associated with very-long chain (A) and polyunsaturated (B & C) triglycerides and diglycerides. Be Beta-regression coefficients of association were calculated by linear regression between genotype and lipid abundance, adjusted for age and sex. P_{slope} was calculated from calculated from simple linear (meta-)regression with 95% confidence intervals. Significantly associated individual metabolites are shown in red. Data from the Fenland cohort only using untargeted lipid profiling.

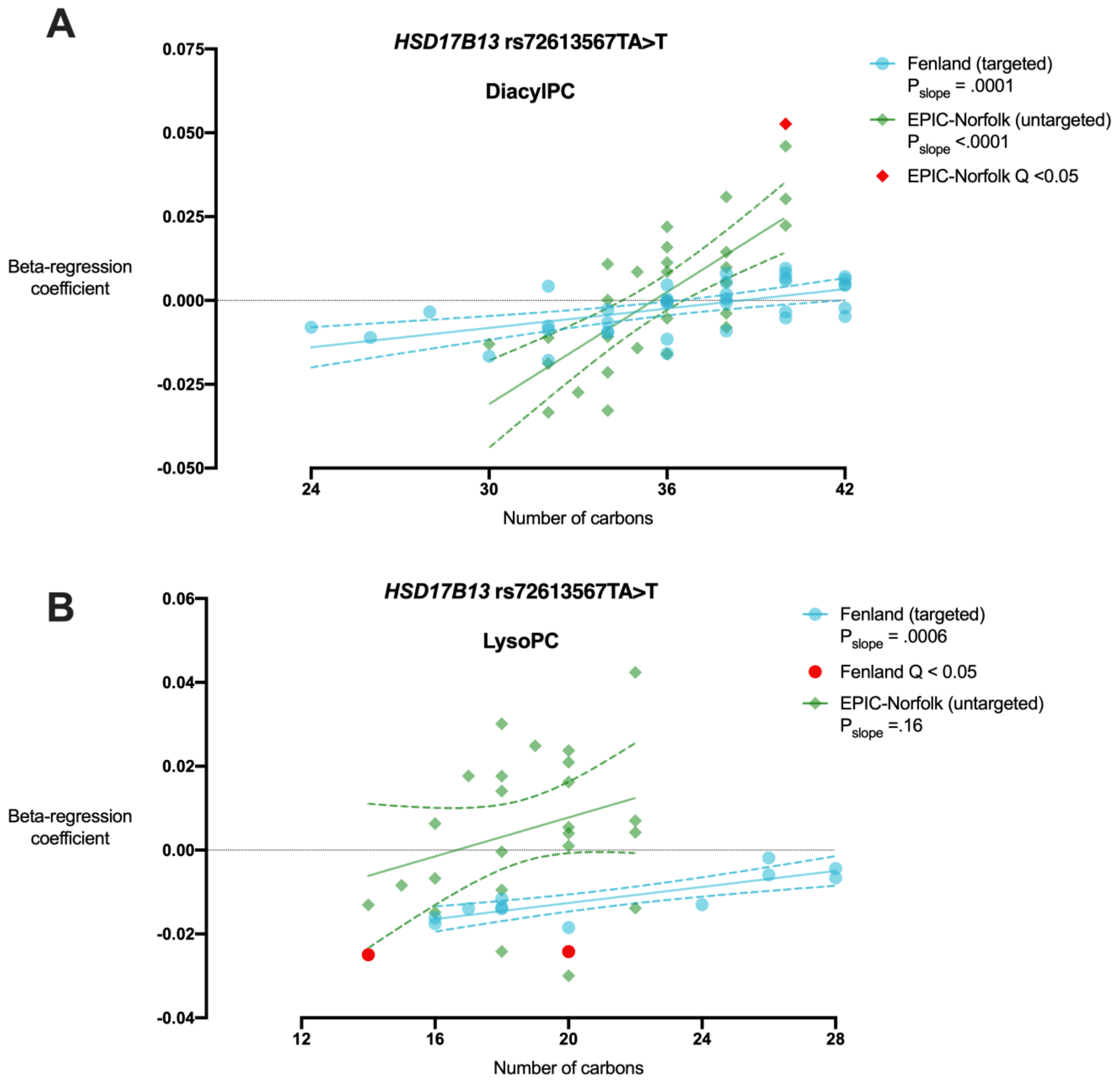

Supplementary Figure 3.

Characteristics of plasma lipid species associated with rs72913567TA>T in *HSD17B13*. The variant was more positively associated with long chain diacylphosphatidylcholines (diacylPC, A) and more negatively associated with short chain lysophosphatidylcholines (lysoPC, B). Beta-regression coefficients of association were calculated by linear regression between genotype and lipid abundance, adjusted for age and sex. P_{slope} was calculated from simple linear (meta-)regression with 95% confidence intervals. Significantly associated individual metabolites are shown in red. Data from the Fenland cohort using targeted lipid profiling and from the EPIC-Norfolk cohort using untargeted metabolite profiling.

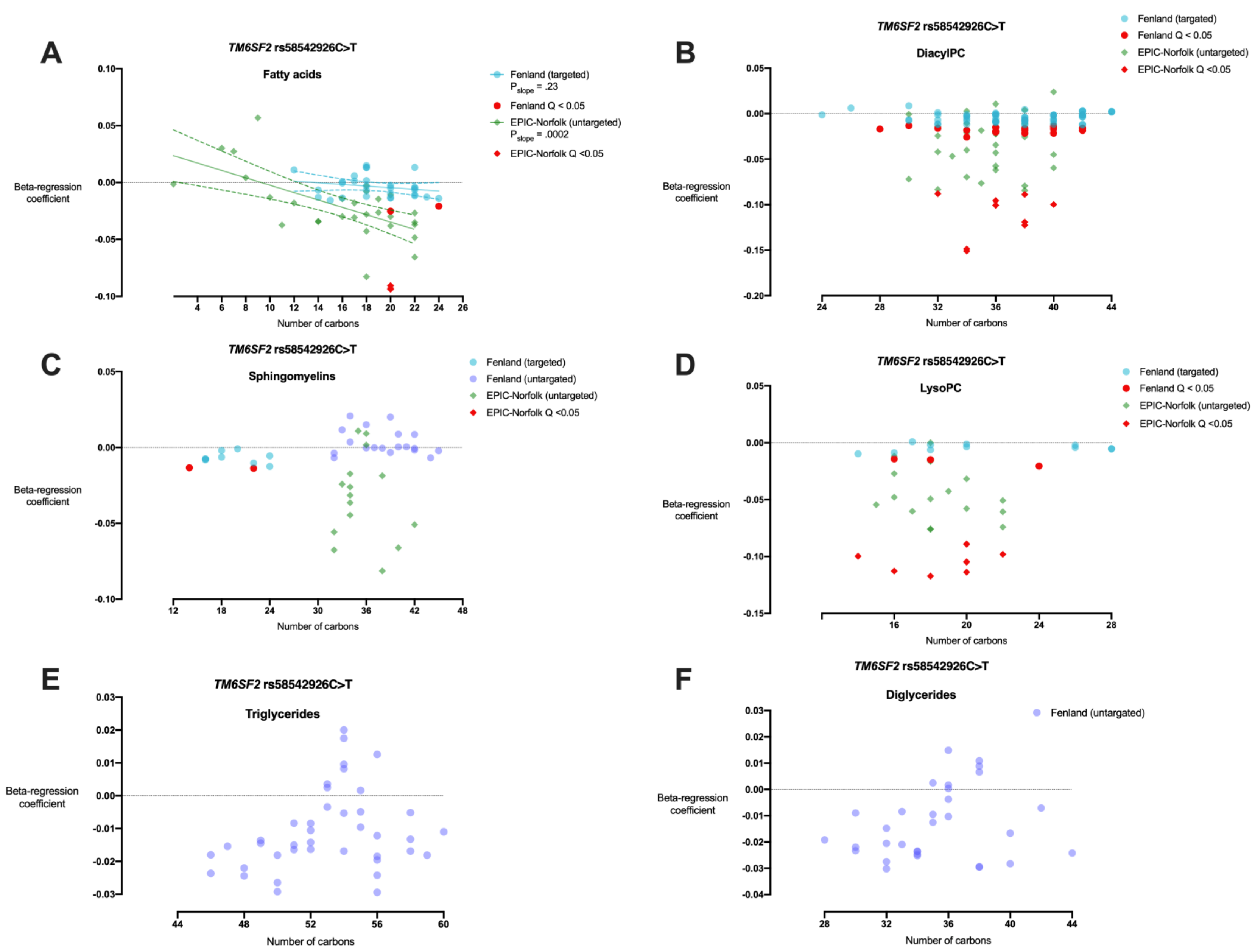

Supplementary Figure 4.

Characteristics of plasma lipid species associated with rs58542926C>T near *TM6SF2*. The variant was negatively associated with a range of lipid species across multiple different metabolomics platforms and cohorts, including fatty acids (A), diacylphosphatidylcholines (diacylPC, B), sphingomyelins (C), and lysophosphatidylcholines (lysoPC, D). Untargeted

lipidomics showed a trend of lower triglycerides (E) and diglycerides (F) with this variant, though no individual lipid species was significantly associated at Q<0.05. Beta-regression coefficients of association were calculated by linear regression between genotype and lipid abundance, adjusted for age and sex. P_{slope} was calculated from calculated from simple linear (meta-)regression with 95% confidence intervals. Significantly associated individual metabolites are shown in red. Data from the Fenland cohort using un-/targeted lipid profiling and from the EPIC-Norfolk cohort using untargeted metabolite profiling.

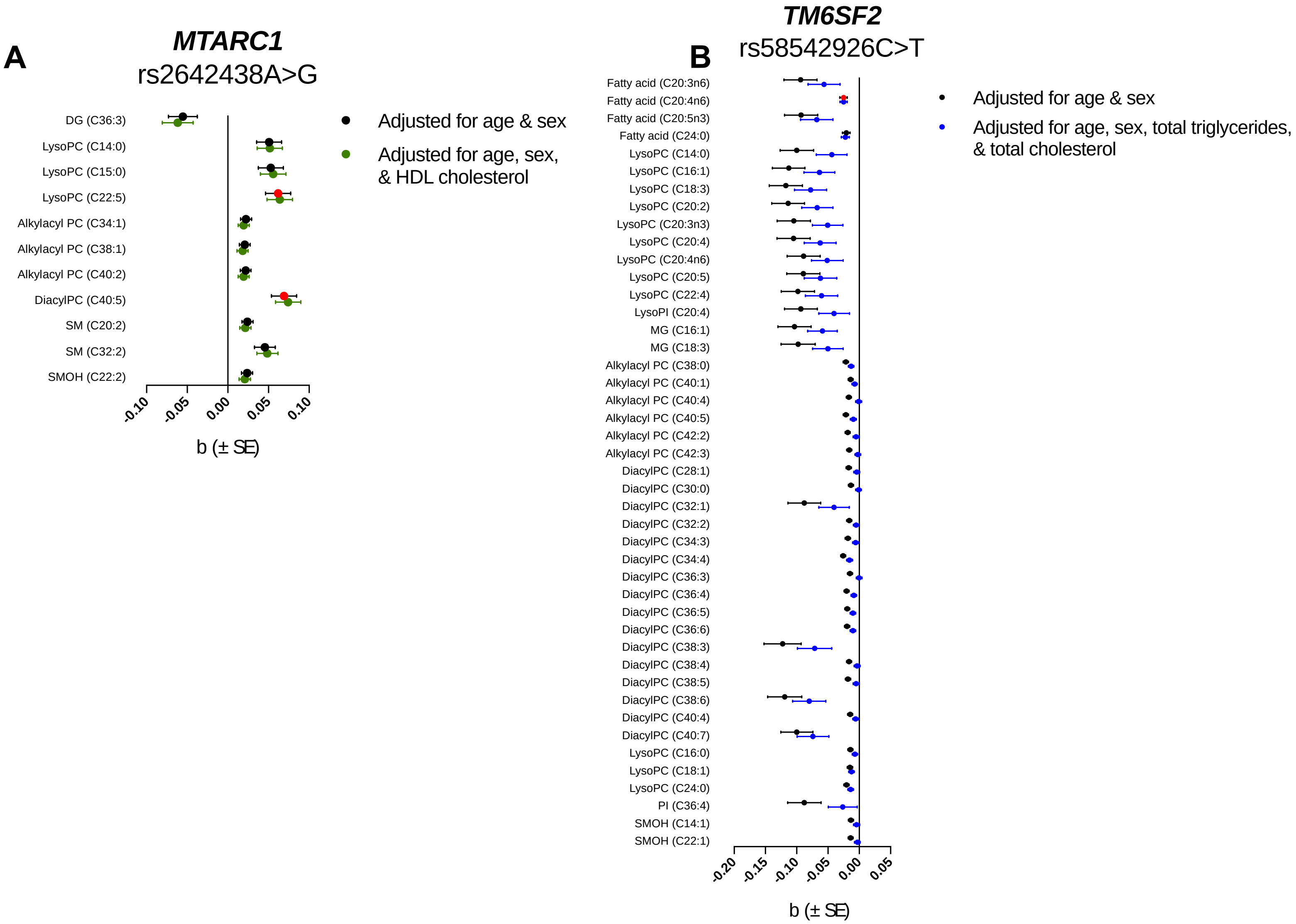

Supplementary Figure 5.

Effect of adjusting for total serum lipids on individual lipid-variant associations. Adjusting for high density lipoprotein (HDL) cholesterol (in addition to age and sex) had minimal effect on the magnitude of variant-metabolite associations for rs2642438A>G in *MTARC1* (A). Adjusting for total cholesterol and triglycerides attenuated the magnitude of change for lipids associated with rs58542926C>T near TM6SF2 (B). Beta-regression coefficients of association were calculated by linear regression between genotype and lipid abundance. All metabolites in black were significantly associated with their respective variants (Q<0.05); the one variant in red remained significantly associated after additional adjustments. Data from targeted and untargeted metabolite profiling in the Fenland and EPIC-Norfolk cohorts.

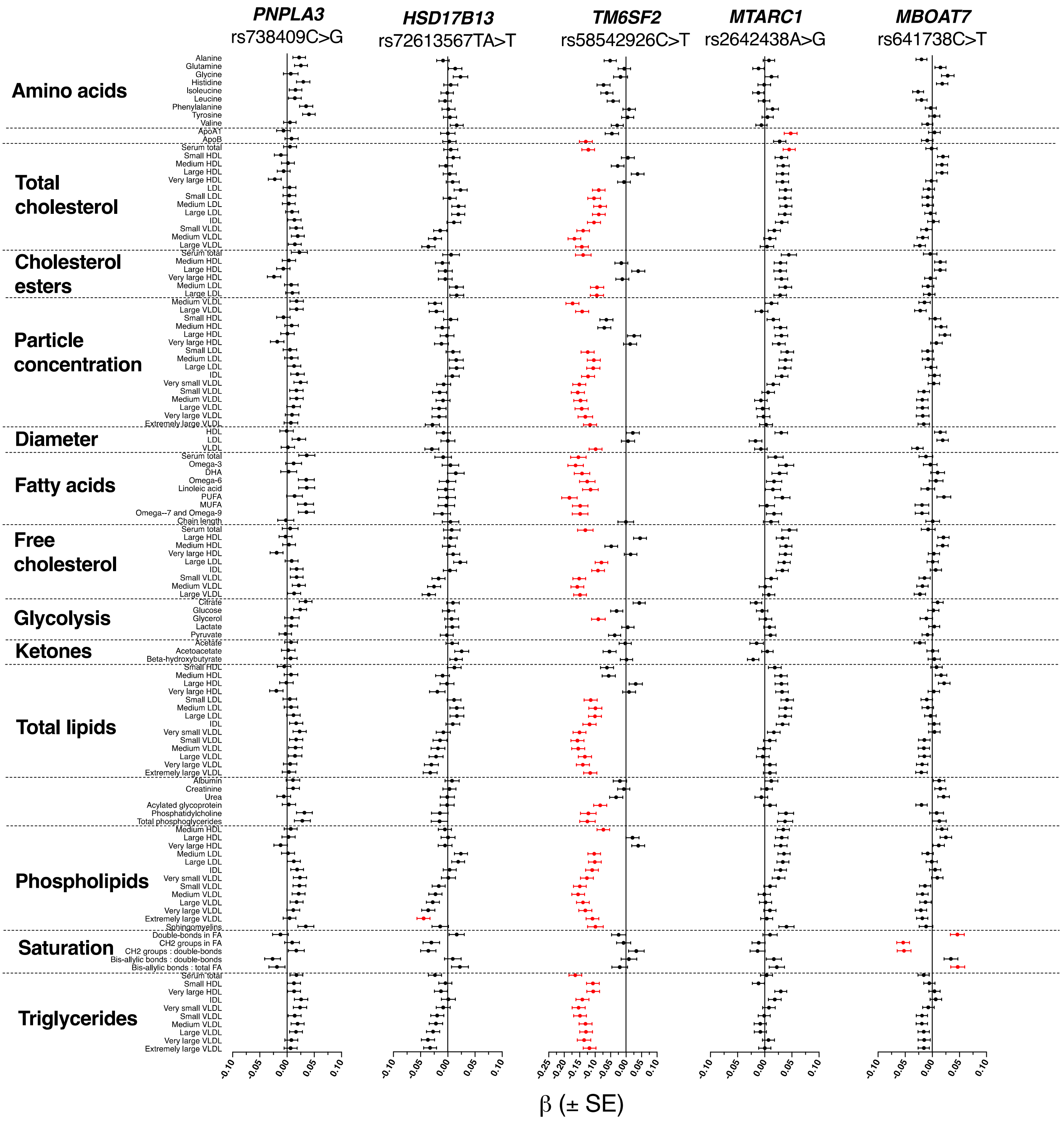

Supplementary Figure 6.

Association (β (± 95% CI)) of lipoparticles and metabolites with NASH-associated SNPs using data from Kettunen *et al*. Metabolites in red have p<0.0004.

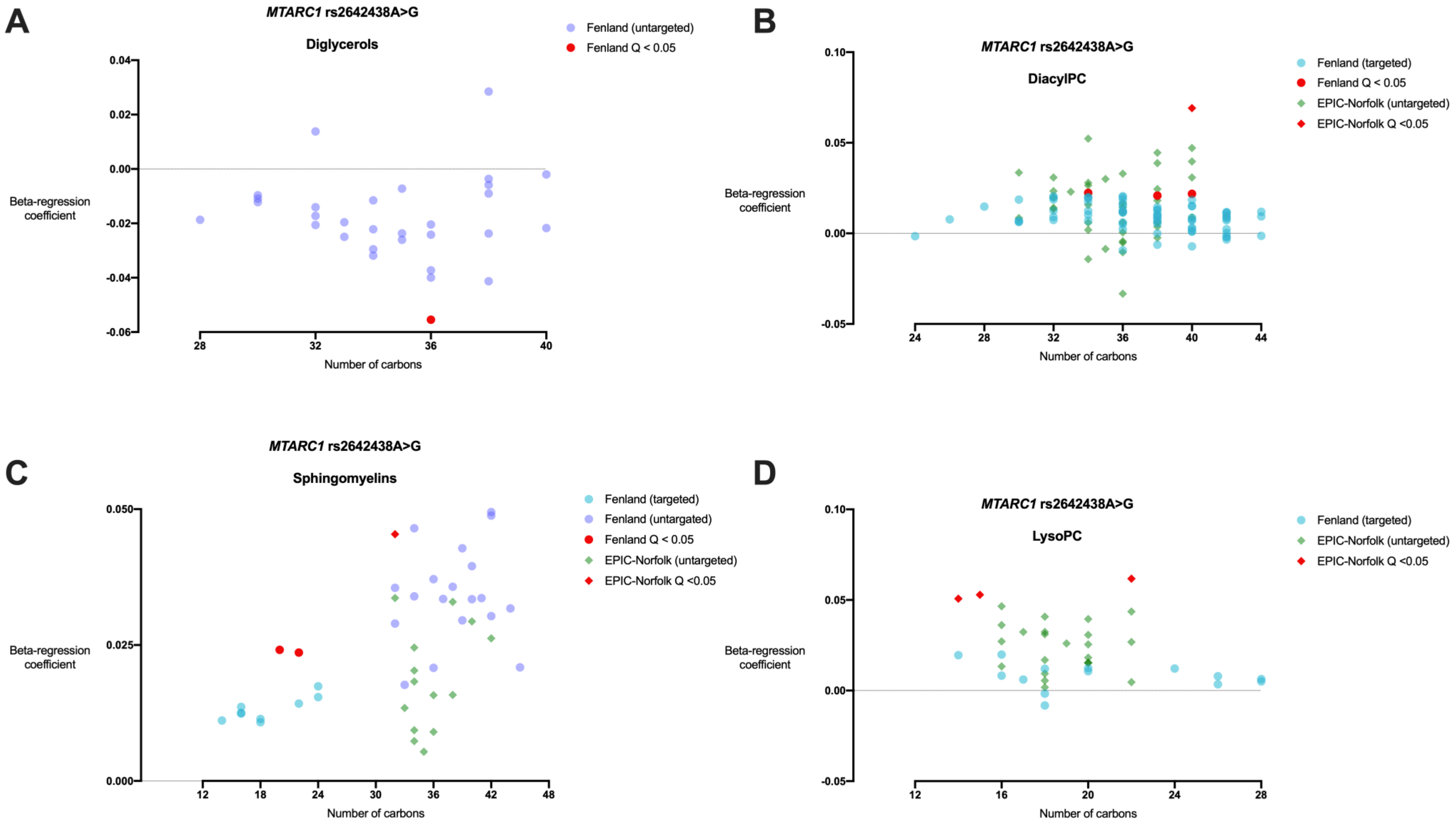

Supplementary Figure 7.

Characteristics of plasma lipid species associated with rs2642438A>G in *MTARC1*. The variant was negatively associated diglycerides (A) but positively associated with other classes of lipid, including diacylphosphatidylcholines (diacylPC, B), sphingomyelins (C), and lysophosphatidylcholines (lysoPC, D). Beta-regression coefficients of association were calculated by linear regression between genotype and lipid abundance, adjusted for age and sex. Significantly associated individual metabolites are shown in red. Data from the Fenland cohort using un-/targeted lipid profiling and from the EPIC-Norfolk cohort using untargeted metabolite profiling.

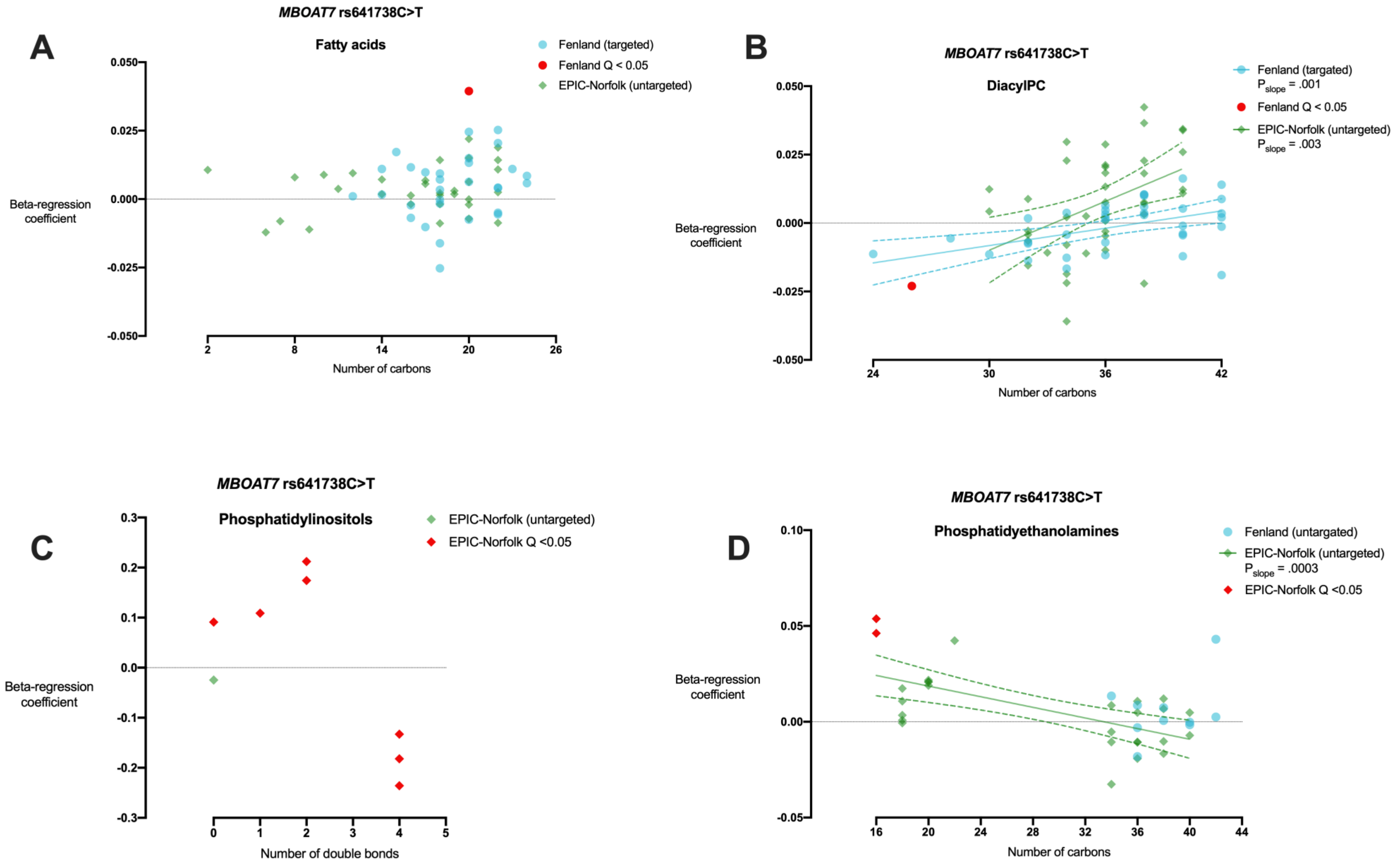

Supplementary Figure 8.

Characteristics of plasma lipid species associated with rs641738C>T near *MBOAT7*. The variant showed a heterogeneous pattern of assocations with fatty acids (A) but there was a consistent positive relationship between carbon chain length and beta-regression coefficient for diacylphosphatidylcholines (diacylPC, B). rs641738C>T showed a strong positive association with

phosphatidylinositols containing 0-2 double-bond chains and negative association with PI containing 4 double bonds (C). A similar negative relationship with carbon chain length was observed with phosphatidylethanolamines (D). Beta-regression coefficient of association were calculated by linear regression between genotype and lipid abundance, adjusted for age and sex. P_{slope} was calculated from simple linear (meta-)regression with 95% confidence intervals. Significantly associated individual metabolites are shown in red. Data from the Fenland cohort using un-/targeted lipid profiling and from the EPIC-Norfolk cohort using untargeted metabolite profiling.

Supplementary Tables

Group	Gene	Variant	Protein consequence	Effect on liver disease	T2DM	CVD	Evidence	Refs
NASH-cirrhosis variants	*PNPLA3*	rs738409 C>G	p.Ile148Met	Harmful	↑	↓	Genome-wide significant variant for liver fat, ALT, and T2DM. Multiple independent replication studies associating with NAFLD (diagnosis), NASH, severity of steatosis, fibrosis, and hepatocellular carcinoma.	(1–8)
	TM6SF2	rs58542926 C>T	p.Glu167Lys	Harmful	↑	↓	Genome-wide significant variant for liver fat, ALT, and T2DM . Multiple independent replication studies associating with NAFLD (diagnosis), NASH, severity of steatosis, fibrosis, and hepatocellular carcinoma.	(5–7,9–11)
	TMC4-MBOAT7	rs641738 C>T	TMC4: p.Gly17Glu MBOAT7: ?	Harmful	↔	↑↔	Genome-wide significant variant for alcoholic cirrhosis. Implicated in NASH, fibrosis, and HCC in multiple candidate gene studies, recently summarised in a meta-analysis. No effect on T2DM but associated with increased risk of stroke.	(12–15)
	HSD17B13	rs72613567 TA>T	p.? (null)	Harmful	↓↔	↔	Genome-wide significant variant for ALT, alcohol-related cirrhosis, and NAFLD cirrhosis. Replicated in several independent cohorts with histological confirmation. Borderline effect on lower risk of T2DM. rs72613567-TA variant results in a splice variant that is degraded, whereas the rs72613567-T variant results in higher expression of HSD17B13.	(7,16–20)
	MTARC1	rs2642438 A>G	p.Thr165Ala	Harmful	↓↔	↔	Recently identified variant associated with higher risk of alcohol-related cirrhosis and NAFLD cirrhosis, as well as higher ALT and higher liver fat across multiple cohorts. Supporting histological evidence from one cohort of adults. Borderline effect on lower risk of T2DM.	(7,21)
Liver fat variants	*GCKR*	rs780094 C>T	Intronic	Harmful	↓	↑	Genome-wide significant variant for liver fat as well as multiple other metabolic traits including triglycerides and insulin resistance. Not associated with ALT or cirrhosis in recent analyses (Abul-Husn et al. and Emdin et al.).	(7,9,16,22–24)
	PPP1R3B	rs4240624 G>A	Intronic	Harmful	↓↔	↑↔	Genome-wide significant variant for liver fat. Not associated with cirrhosis in recent analyses (Abul-Husn et al. and Emdin et al.).	(7,9,16,22,25)
	LYPLAL1	rs12137855 T>C	Intergenic	Harmful	↓	↑↔	Genome-wide significant variant for liver fat. Not associated with ALT or cirrhosis in recent analyses (Abul-Husn et al. and Emdin et al.).	(7,9,16,25–27)
Metabolic cirrhosis	*HFE*	rs1800562 G>A	p.Cys282Tyr	Harmful	↑↔	↔	Most common mutation associated with hereditary haemochromatosis and implicated in all-cause cirrhosis.	(7,28–32)
	SERPINA1	rs28929474 C>T	p.Glu366Lys	Harmful	↔	↔	Most common mutation associated with alpha-1-antitrysin deficiency. Genome-wide significant variant for ALT and implicated in all-cause cirrhosis.	(7,16,32,33)

Supplementary Table 1.

Summary of evidence for selection of variants included in the study. T2DM, type 2 diabetes mellitus; CVD, cardiovascular disease.

	Steatosis	Without steatosis	Q-value
	n=2301	n=6834	
**Age (years)**	50.3 ±7.1	48.5 ±7.5	7.5E-30
**Male sex, n (%)**	1346 (59)	2859 (42)	5.4E-48
**European descent, n (%)**	2301 (100)	6834 (100)	-
**BMI, kg/m^2**	30.5 ±5.0	25.3 ±3.7	<1.0E-300
**Waist-to-hip ratio**	0.94 ±0.1	0.85 ±0.1	<1.0E-300
**Body fat percentage (%)**	37.1 ±7.5	32.0 ±7.4	<1.0E-300
**Systolic blood pressure (mmHg)**	127 ±15	119 ±15	3.1E-87
**Diastolic blood pressure (mmHg)**	78 ±10	72 ±10	6.7E-157
**T2DM, n (%)**	147 (6.4)	72 (1.1)	2.0E-68
**IFG, n (%)**	43 (1.9)	13 (0.2)	4.5E-19
**High alcohol consumption, n (%)**	13 (0.6)	26 (0.4)	.18
**Total cholesterol, mg/dL**	5.6 ±1.1	5.4 ±1.	1.5E-15
**LDL cholesterol, mg/dL**	3.6 ±0.9	3.3 ±0.9	4.3E-18
**HDL cholesterol, mg/dL**	1.4 ±0.4	1.6 ±0.4	4.7E-126
**Triglycerides, mg/dL**	1.6 ±1.3	1.0 ±0.7	1.5E-134
**NEFA, µmol/L**	347 ±158	339 ±167	.002
**Albumin, g/L**	41.6 ±2.7	41.7 ±2.6	4.2E-08
**ALP, IU/L**	89.7 ±23	83.7 ±24	1.4E-22
**ALT, IU/L**	35.8 ±20	26.2 ±14	1.3E-127
**Bilirubin, µmol/L**	10.0 ±5.6	10.6 ±5.5	2.4E-15
**GGT, IU/L**	45.2 ±47	31.0 ±30	7.0E-52
**Creatinine, µmol/L**	80 ±16	76 ±15	.003
**Urea, µmol/L**	5.2 ±1.3	5.0 ±1.2	.92
**Glucose (fasting), mmol/L**	5.1 ±0.9	4.7 ±0.5	4.9E-109
**Glucose (120min), mmol/L**	6.1 ±2.1	5.0 ±1.4	6.7E-175
**Insulin (fasting), pmol/L**	69.7 ±48.1	38.7 ±22.5	<1.0E-300
**HbA1c, mmol/mol**	38.8 ±6.0	36.7 ±4.0	7.8E-25
**HOMA-IR**	2.3 ±1.9	1.2 ±0.8	<1.0E-300
**ADIPO-IR**	24.1 ±19.9	13.0 ±10.5	8.4E-252

Supplementary table 2.

Baseline characteristics of participants with and without hepatic steatosis. Data represented as mean ± SD for continuous variables and number (%) for categorical variables. Characteristics were compared between groups using regression adjusting for age and sex. Data from the Fenland cohort.

Supplementary Table 3 [Excel spreadsheet].

Results of all metabolite-associations with gene variants, polygenic gene scores, and steatosis from the Fenland Study and EPIC-Norfolk cohorts.

	PNPLA3 rs738409			
	**CC**	**CG**	**GG**	**Q-value**
	n=6605	n=3791	n=538	
**Age (years)**	48.6 ±7.5	48.6 ±7.5	48.4 ±7.6	.92
**Male sex, n (%)**	3084 (46.7)	1790 (47.2)	237 (44.1)	.92
**European descent, n (%)**	6605 (100)	3791 (100)	538 (100)	-
**BMI, kg/m2**	26.9 ±4.8	26.9 ±4.8	26.7 ±4.8	.92
**Waist-to-hip ratio**	0.9 ±0.1	0.9 ±0.1	0.9 ±0.1	.86
**Body fat percentage (%)**	33.5 ±7.9	33.5 ±7.8	34. ±7.8	.83
**Systolic blood pressure (mmHg)**	121 ±15	122 ±15	121 ±15	.92
**Diastolic blood pressure (mmHg)**	74 ±10	74 ±10	74 ±11	.44
**Hepatic steatosis, n (%)**	1280 (23.2)	884 (28)	137 (29.7)	.0002
**T2DM, n (%)**	172 (2.6)	93 (2.5)	15 (2.8)	.93
**IFG, n (%)**	42 (0.6)	22 (0.6)	6 (1.1)	.82
**High alcohol consumption, n (%)**	31 (0.5)	21 (0.6)	3 (0.6)	.95
**Total cholesterol, mg/dL**	5.4 ±1.	5.4 ±1.	5.5 ±1.	.92
**LDL cholesterol, mg/dL**	3.4 ±0.9	3.4 ±0.9	3.4 ±0.9	.76
**HDL cholesterol, mg/dL**	1.5 ±0.4	1.5 ±0.4	1.6 ±0.4	.17
**Triglycerides, mg/dL**	1.2 ±0.9	1.2 ±0.8	1.2 ±0.8	.64
**NEFA, µmol/L**	338.8 ±161.5	344.3 ±168.	346.7 ±166.8	.27
**Albumin, g/L**	41.7 ±2.7	41.7 ±2.6	41.9 ±2.6	.31
**ALP, IU/L**	84.4 ±24.	84.8 ±23.7	84.9 ±24.7	.61
**ALT, IU/L**	28.3 ±15.6	30. ±17.8	32.5 ±21.6	2.1E-13
**Bilirubin, µmol/L**	10.5 ±5.7	10.5 ±5.4	10.8 ±5.2	.52
**GGT, IU/L**	34.8 ±33.9	34.8 ±33.3	38. ±68.7	.38
**Creatinine, µmol/L**	77. ±15.6	77.5 ±15.6	76.5 ±15.1	.49
**Urea, µmol/L**	5.1 ±1.3	5.1 ±1.3	5. ±1.2	.92
**Glucose (fasting), mmol/L**	4.8 ±0.6	4.8 ±0.7	4.8 ±0.6	.92
**Glucose (120min), mmol/L**	5.3 ±1.7	5.3 ±1.7	5.3 ±1.7	.92
**Insulin (fasting), pmol/L**	47.4 ±35.2	48.5 ±35.4	49.5 ±37.9	.21
**HbA1c, mmol/mol**	37.3 ±4.8	37.1 ±4.6	36.7 ±3.4	.17
**HOMA-IR**	1.5 ±1.3	1.6 ±1.4	1.6 ±1.6	.16
**ADIPO-IR**	16. ±14.6	16.7 ±15.2	16.6 ±14.7	.18

Supplementary table 4.

Baseline characteristics stratified by PNPLA3 (rs738409) genotype. Data represented as mean ± SD for continuous variables and number (%) for categorical variables. Characteristics were compared between groups using regression using an additive model for age, sex, and principal genetic components. Data from the Fenland cohort.

	TM6SF2 rs58542926			
	**CC**	**CT**	**TT**	**Q-value**
	n=9281	n=1571	n=82	
**Age (years)**	48.6 ±7.5	48.7 ±7.4	48.7 ±7.1	.92
**Male sex, n (%)**	4322 (46.6)	747 (47.6)	42 (51.2)	.61
**European descent, n (%)**	9281 (100)	1571 (100)	82 (100)	-
**BMI, kg/m2**	26.9 ±4.8	26.9 ±4.8	26.7 ±4.3	.92
**Waist-to-hip ratio**	0.9 ±0.1	0.9 ±0.1	0.9 ±0.1	.4
**Body fat percentage (%)**	33.6 ±7.9	33.5 ±7.8	33.9 ±7.7	.76
**Systolic blood pressure (mmHg)**	122 ±15	122 ±15	125 ±19	.61
**Diastolic blood pressure (mmHg)**	74 ±10	74 ±10	75 ±13	.14
**Hepatic steatosis, n (%)**	1892 (24.5)	386 (29.)	29 (32.4)	.002
**T2DM, n (%)**	234 (2.5)	42 (2.7)	4 (4.9)	.69
**IFG, n (%)**	61 (0.7)	8 (0.5)	1 (1.2)	.92
**High alcohol consumption, n (%)**	47 (0.5)	8 (0.5)	0	.68
**Total cholesterol, mg/dL**	5.5 ±1.	5.3 ±1.	4.8 ±1.1	5.1E-14
**LDL cholesterol, mg/dL**	3.4 ±0.9	3.3 ±0.9	2.9 ±0.9	2.5E-12
**HDL cholesterol, mg/dL**	1.5 ±0.4	1.5 ±0.4	1.5 ±0.5	.23
**Triglycerides, mg/dL**	1.2 ±0.9	1.1 ±0.7	0.9 ±0.6	3.7E-07
**NEFA, µmol/L**	343 ±164	332 ±164	334 ±155	.16
**Albumin, g/L**	41.7 ±2.6	41.9 ±2.7	41.7 ±2.6	.07
**ALP, IU/L**	84.9 ±24.3	82.7 ±22.	85.7 ±19.9	.01
**ALT, IU/L**	28.9 ±16.7	30. ±17.1	33.9 ±19.	.01
**Bilirubin, µmol/L**	10.5 ±5.6	10.7 ±5.3	11.2 ±6.	.26
**GGT, IU/L**	35. ±36.5	34.8 ±34.6	36.4 ±31.4	.92
**Creatinine, µmol/L**	77.1 ±15.5	77.4 ±15.9	78.2 ±17.9	.92
**Urea, µmol/L**	5.1 ±1.3	5.1 ±1.3	4.9 ±1.3	.34
**Glucose (fasting), mmol/L**	4.8 ±0.6	4.9 ±0.8	4.9 ±0.6	.27
**Glucose (120min), mmol/L**	5.3 ±1.7	5.3 ±1.7	5.2 ±1.6	.52
**Insulin (fasting), pmol/L**	47.8 ±35.4	48.1 ±35.5	50.1 ±30.5	.92
**HbA1c, mmol/mol**	37.2 ±4.7	37.4 ±4.7	37.7 ±4.1	.61
**HOMA-IR**	1.5 ±1.3	1.5 ±1.3	1.6 ±1.	.98
**ADIPO-IR**	16.4 ±14.9	15.6 ±14.1	16.9 ±16.1	.35

Supplementary table 5.

Baseline characteristics stratified by TM6SF2 (rs58542926) genotype. Data represented as mean ± SD for continuous variables and number (%) for categorical variables. Characteristics were compared between groups using using an additive model adjusting for age, sex, and principal genetic components. Data from the Fenland cohort.

	MBOAT7 rs641738			
	**CC**	**CT**	**TT**	**Q-value**
	n=3418	n=5494	n=2022	
**Age (years)**	49 ±7.5	49 ±7.5	49 ±7.4	.92
**Male sex, n (%)**	1654 (48)	2522 (46)	935 (46)	.2
**European descent, n (%)**	3418 (100)	5494 (100)	2022 (100)	-
**BMI, kg/m2**	26.9 ±4.9	26.9 ±4.8	27 ±4.8	.89
**Waist-to-hip ratio**	0.9 ±0.1	0.9 ±0.1	0.9 ±0.1	.92
**Body fat percentage (%)**	33.5 ±7.9	33.5 ±7.8	33.7 ±7.9	.98
**Systolic blood pressure (mmHg)**	122 ±15	122 ±15	122 ±15	.73
**Diastolic blood pressure (mmHg)**	74 ±10	74 ±10	74 ±10	.95
**Hepatic steatosis, n (%)**	707 (25)	1144 (25)	450 (26)	.26
**T2DM, n (%)**	95 (2.8)	129 (2.4)	56 (2.8)	.92
**IFG, n (%)**	23 (0.7)	35 (0.6)	12 (0.6)	.92
**High alcohol consumption, n (%)**	20 (0.6)	26 (0.5)	9 (0.5)	.92
**Total cholesterol, mg/dL**	5.4 ±1.1	5.4 ±1.	5.4 ±1.	.92
**LDL cholesterol, mg/dL**	3.4 ±0.9	3.4 ±0.9	3.4 ±0.9	.39
**HDL cholesterol, mg/dL**	1.5 ±0.4	1.5 ±0.4	1.5 ±0.4	.27
**Triglycerides, mg/dL**	1.2 ±0.9	1.2 ±0.8	1.2 ±1.1	.27
**NEFA, µmol/L**	346 ±169	338 ±162	340 ±161	.23
**Albumin, g/L**	41.8 ±2.7	41.7 ±2.6	41.8 ±2.6	.86
**ALP, IU/L**	83.6 ±23.8	84.5 ±23.4	86.3 ±25.5	.0001
**ALT, IU/L**	29.4 ±16.2	28.8 ±17.1	29.4 ±16.7	.92
**Bilirubin, µmol/L**	10.6 ±5.5	10.4 ±5.6	10.4 ±5.6	.67
**GGT, IU/L**	36. ±41.1	34.2 ±31.8	35.3 ±38.2	.76
**Creatinine, µmol/L**	77.3 ±15.8	77.1 ±15.3	77. ±15.8	.77
**Urea, µmol/L**	5.1 ±1.3	5.1 ±1.2	5.0 ±1.3	.69
**Glucose (fasting), mmol/L**	4.8 ±0.7	4.8 ±0.6	4.9 ±0.8	.16
**Glucose (120min), mmol/L**	5.3 ±1.8	5.3 ±1.7	5.3 ±1.8	.98
**Insulin (fasting), pmol/L**	48.8 ±36.4	47.3 ±34.9	47.9 ±35.1	.68
**HbA1c, mmol/mol**	37.4 ±5.	37.1 ±4.5	37.3 ±4.7	.86
**HOMA-IR**	1.6 ±1.3	1.5 ±1.3	1.6 ±1.5	.94
**ADIPO-IR**	16.9 ±15.8	15.9 ±14.4	16.3 ±14.3	.26

Supplementary table 6.

Baseline characteristics stratified by MBOAT7 (rs641738) genotype. Data represented as mean ± SD for continuous variables and number (%) for categorical variables. Characteristics were compared between groups using an additive model adjusting for age, sex, and principal genetic components. Data from the Fenland cohort.

	HSD17B13 rs72613567			
	**TA-TA**	**T-TA**	T-T	**Q-value**
	n=828	n=4605	n=6023	
**Age (years)**	48.4 ±7.5	48.6 ±7.5	48.6 ±7.5	.92
**Male sex, n (%)**	393 (49.3)	2079 (47.3)	2639 (46)	.17
**European descent, n (%)**	828 (100)	4605 (100)	6023 (100)	-
**BMI, kg/m2**	26.6 ±5.	26.9 ±4.7	26.9 ±4.9	.27
**Waist-to-hip ratio**	0.9 ±0.1	0.9 ±0.1	0.9 ±0.1	.86
**Body fat percentage (%)**	32.8 ±7.7	33.5 ±7.9	33.7 ±7.9	.34
**Systolic blood pressure (mmHg)**	122.1 ±15.9	122. ±15.4	121.8 ±15.3	.98
**Diastolic blood pressure (mmHg)**	73.8 ±10.6	73.8 ±10.2	74. ±10.1	.39
**Hepatic steatosis, n (%)**	159 (24.4)	918 (25)	1224 (25.5)	.63
**T2DM, n (%)**	21 (2.6)	109 (2.5)	150 (2.6)	.92
**IFG, n (%)**	7 (0.9)	28 (0.6)	35 (0.6)	.8
**High alcohol consumption, n (%)**	2 (0.3)	25 (0.6)	28 (0.5)	.1
**Total cholesterol, mg/dL**	5.4 ±1.	5.4 ±1.	5.4 ±1.	.86
**LDL cholesterol, mg/dL**	3.4 ±0.9	3.4 ±0.9	3.4 ±0.9	.92
**HDL cholesterol, mg/dL**	1.5 ±0.4	1.5 ±0.4	1.5 ±0.4	.89
**Triglycerides, mg/dL**	1.2 ±0.8	1.2 ±0.8	1.2 ±1.	.92
**NEFA, µmol/L**	330.3 ±160	341.2 ±165	342.6 ±164	.54
**Albumin, g/L**	41.8 ±2.6	41.7 ±2.7	41.7 ±2.6	.86
**ALP, IU/L**	85.2 ±24.6	85.0 ±24.8	84.1 ±23.2	.18
**ALT, IU/L**	28.8 ±16.0	28.5 ±15.5	29.6 ±17.7	.001
**Bilirubin, µmol/L**	10.5 ±5.4	10.5 ±5.5	10.5 ±5.7	.92
**GGT, IU/L**	37.8 ±73.1	35.1 ±33.2	34.5 ±30.1	.2
**Creatinine, µmol/L**	77. ±15.7	77.3 ±15.4	77. ±15.6	.75
**Urea, µmol/L**	5. ±1.2	5.1 ±1.3	5.1 ±1.3	.25
**Glucose (fasting), mmol/L**	4.9 ±0.7	4.8 ±0.7	4.8 ±0.7	.63
**Glucose (120min), mmol/L**	5.2 ±1.8	5.3 ±1.8	5.3 ±1.7	.86
**Insulin (fasting), pmol/L**	47.1 ±37.6	47.5 ±34.5	48.3 ±35.8	.33
**HbA1c, mmol/mol**	37.6 ±6.7	37.3 ±4.6	37.1 ±4.4	.16
**HOMA-IR**	1.5 ±1.3	1.5 ±1.3	1.5 ±1.4	.43
**ADIPO-IR**	15.5 ±15.7	16.2 ±14.9	16.4 ±14.6	.36

Supplementary table 7.

Baseline characteristics stratified by HSD17B13 (rs72613567) genotype. Data represented as mean ± SD for continuous variables and number (%) for categorical variables. Characteristics were compared between groups using an additive model adjusting for age, sex, and principal genetic components. Data from the Fenland cohort.

	MTARC1 rs2642438			
	**AA**	**GA**	**GG**	**Q-value**
	n=819	n=4358	n=5081	
**Age (years)**	48.9 ±7.4	48.5 ±7.5	48.7 ±7.5	.92
**Male sex, n (%)**	401 (45.6)	2139 (46.1)	2571 (47.5)	.27
**European descent, n (%)**	819 (100)	4358 (100)	5081 (100)	-
**BMI, kg/m2**	26.9 ±4.8	26.9 ±4.8	26.9 ±4.8	.76
**Waist-to-hip ratio**	0.9 ±0.1	0.9 ±0.1	0.9 ±0.1	.33
**Body fat percentage (%)**	33.8 ±7.8	33.6 ±7.9	33.5 ±7.8	.92
**Systolic blood pressure (mmHg)**	121.6 ±15.3	121.7 ±15.2	122.1 ±15.5	.64
**Diastolic blood pressure (mmHg)**	73.5 ±10.1	73.9 ±10.1	74. ±10.3	.62
**Hepatic steatosis, n (%)**	185 (25.)	993 (25.8)	1123 (24.7)	.99
**T2DM, n (%)**	22 (2.5)	123 (2.7)	135 (2.5)	.92
**IFG, n (%)**	5 (0.6)	28 (0.6)	37 (0.7)	.86
**High alcohol consumption, n (%)**	2 (0.2)	25 (0.5)	28 (0.5)	.86
**Total cholesterol, mg/dL**	5.4 ±1.0	5.4 ±1.1	5.4 ±1.0	.76
**LDL cholesterol, mg/dL**	3.4 ±0.9	3.4 ±0.9	3.4 ±0.9	.92
**HDL cholesterol, mg/dL**	1.51 ±0.4	1.52 ±0.4	1.54 ±0.4	.002
**Triglycerides, mg/dL**	1.19 ±0.8	1.21 ±1.0	1.17 ±0.8	.16
**NEFA, µmol/L**	347 ±175	335 ±160	345 ±165	.2
**Albumin, g/L**	41.6 ±2.6	41.7 ±2.7	41.8 ±2.7	.03
**ALP, IU/L**	85.2 ±23	84.2 ±24	84.8 ±24	.94
**ALT, IU/L**	27.9 ±14	28.7 ±16	29.5 ±18	.02
**Bilirubin, µmol/L**	10.5 ±5.8	10.4 ±5.4	10.5 ±5.7	.86
**GGT, IU/L**	34.3 ±30.2	34.9 ±35.9	35.1 ±37.3	.92
**Creatinine, µmol/L**	76.6 ±14.6	76.9 ±15.5	77.4 ±15.8	.45
**Urea, µmol/L**	5.1 ±1.2	5.1 ±1.3	5.1 ±1.3	.92
**Glucose (fasting), mmol/L**	4.8 ±0.8	4.8 ±0.6	4.8 ±0.7	.92
**Glucose (120min), mmol/L**	5.3 ±1.8	5.3 ±1.7	5.3 ±1.7	.35
**Insulin (fasting), pmol/L**	48. ±40.6	48. ±35.1	47.7 ±34.8	.76
**HbA1c, mmol/mol**	37.0 ±3.9	37.1 ±4.7	37.4 ±4.8	.27
**HOMA-IR**	1.5 ±1.5	1.5 ±1.3	1.5 ±1.3	.86
**ADIPO-IR**	16.1 ±14.6	16.1 ±15.1	16.4 ±14.6	.73

Supplementary table 8.
Baseline characteristics stratified by MTARC1 (rs2642438) genotype. Data represented as mean ± SD for continuous variables and number (%) for categorical variables. Characteristics were compared between groups using an additive model adjusting for age, sex, and principal genetic components. Data from the Fenland cohort.

Supplementary Table 9 [Excel spreadsheet].
Results of metabolite-associations with gene variants identified using Phenoscanner. Results with P<0.05 are included.

Supplementary references

1. Sookoian, S. and Pirola, C. J. (2011) Meta-analysis of the influence of I148M variant of patatin-like phospholipase domain containing 3 gene (PNPLA3) on the susceptibility and histological severity of nonalcoholic fatty liver disease. *Hepatology*, **53**, 1883–1894.

2. Speliotes, E. K., Butler, J. L., Palmer, C. D., Voight, B. F., GIANT Consortium, MIGen Consortium, NASH Clinical Research Network and Hirschhorn, J. N. (2010) PNPLA3 variants specifically confer increased risk for histologic nonalcoholic fatty liver disease but not metabolic disease. *Hepatology*, **52**, 904–912.

3. Romeo, S., Kozlitina, J., Xing, C., Pertsemlidis, A., Cox, D., Pennacchio, L. a, Boerwinkle, E., Cohen, J. C. and Hobbs, H. H. (2008) Genetic variation in PNPLA3 confers susceptibility to nonalcoholic fatty liver disease. *Nat. Genet.*, **40**, 1461–1465.

4. Kozlitina, J., Smagris, E., Stender, S., Nordestgaard, B. G., Zhou, H. H., Tybjærg-Hansen, A., Vogt, T. F., Hobbs, H. H. and Cohen, J. C. (2014) Exome-wide association study identifies a TM6SF2 variant that confers susceptibility to nonalcoholic fatty liver disease. *Nat. Genet.*, **46**, 352–356.

5. Chambers, J. C., Zhang, W., Sehmi, J., Li, X., Wass, M. N., Van Der Harst, P., Holm, H., Sanna, S., Kavousi, M., Baumeister, S. E., et al. (2011) Genome-wide association study identifies loci influencing concentrations of liver enzymes in plasma. *Nat. Genet.*, **43**, 1131–1138.

6. Mahajan, A., Taliun, D., Thurner, M., Robertson, N. R., Torres, J. M., Rayner, N. W., Payne, A. J., Steinthorsdottir, V., Scott, R. A., Grarup, N., et al. (2018) Fine-mapping type 2 diabetes loci to single-variant resolution using high-density imputation and islet-specific epigenome maps. *Nat. Genet.*, **50**, 1505–1513.

7. Emdin, C. A., Haas, M., Khera, A. V, Aragam, K., Chaffin, M., Klarin, D., Hindy, G., Jian, L., Wei, W.-Q., Feng, Q., et al. (2019) A missense variant in Mitochondrial Amidoxime Reducing Component 1 gene and protection against liver disease. *PLoS Genet.*, **16**, e1008629

8. Singal, A. G., Manjunath, H., Yopp, A. C., Beg, M. S., Marrero, J. A., Gopal, P. and Waljee, A. K. (2014) The effect of PNPLA3 on fibrosis progression and development of hepatocellular carcinoma: a meta-analysis. *Am J Gastroenterol*, **109**, 325–334.

9. Speliotes, E. K., Yerges-armstrong, L. M., Wu, J., Hernaez, R., Lauren, J., Palmer, C. D., Gudnason, V., Eiriksdottir, G., Garcia, M. E., Launer, L. J., et al. (2011) Genome-Wide Association Analysis Identifies Variants Associated with Nonalcoholic Fatty Liver Disease That Have Distinct Effects on Metabolic Traits. *Plos Genet.*, **7**, e1001324.

10. Pirola, C. J. and Sookoian, S. (2015) The dual and opposite role of the TM6SF2-rs58542926 variant in protecting against cardiovascular disease and conferring risk for nonalcoholic fatty liver: A meta-analysis. *Hepatology*, **62**, 1742-1756

11. Stickel, F., Buch, S., Nischalke, H. D., Weiss, K. H., Gotthardt, D., Fischer, J., Rosendahl, J., Marot, A., Elamly, M., Casper, M., et al. (2018) Genetic variants in PNPLA3 and TM6SF2 predispose to the development of hepatocellular carcinoma in individuals with alcohol-related cirrhosis. *Am. J. Gastroenterol.*, **113**, 1475-1483.

12. Buch, S., Stickel, F., Trépo, E., Way, M., Herrmann, A., Nischalke, H. D., Brosch, M., Rosendahl, J., Berg, T., Ridinger, M., et al. (2015) A genome-wide association study confirms PNPLA3 and identifies TM6SF2 and MBOAT7 as risk loci for alcohol-related cirrhosis. *Nat. Genet.*, **47**, 1443–1448.

13. Teo, K., Abeysekera, K. W., Adams, L., Aigner, E., Banerjee, R., Basu, P., Berg, T., Bhatnagar, P., Buch, S., Canbay, A., et al. (2019) rs641738C>T near MBOAT7 promotes steatosis, NASH, fibrosis and hepatocellular carcinoma in non-alcoholic fatty liver disease: a meta-analysis. *medRxiv*, https://doi.org/10.1101/19013623.

14. Mancina, R. M., Dongiovanni, P., Petta, S., Pingitore, P., Meroni, M., Rametta, R., Boren, J., Montalcini, T., Pujia, A., Wiklund, O., et al. (2016) The MBOAT7-TMC4 Variant rs641738 Increases Risk of Nonalcoholic Fatty Liver Disease in Individuals of European Descent. *Gastroenterology*, **150**, 1219-1230e6.

15. Donati, B., Dongiovanni, P., Romeo, S., Meroni, M., McCain, M., Miele, L., Petta, S., Maier, S., Rosso, C., De Luca, L., et al. (2017) MBOAT7 rs641738 variant and hepatocellular carcinoma in non-cirrhotic individuals. *Sci. Rep.*, **7**, 4492.

16. Abul-Husn, N. S., Cheng, X., Li, A. H., Xin, Y., Schurmann, C., Stevis, P., Liu, Y., Kozlitina, J., Stender, S., Wood, G. C., et al. (2018) A Protein-Truncating *HSD17B13* Variant and Protection from Chronic Liver Disease. *N. Engl. J. Med.*, **378**, 1096–1106.

17. Ma, Y., Belyaeva, O. V., Brown, P. M., Fujita, K., Valles, K., Karki, S., de Boer, Y. S., Koh, C., Chen, Y., Du, X., et al. (2018) HSD17B13 is a Hepatic Retinol Dehydrogenase Associated with Histological Features of Non-Alcoholic Fatty Liver Disease. *Hepatology*, **69**, 1504-1519

18. Adam, M., Heikelä, H., Sobolewski, C., Portius, D., Mäki-Jouppila, J., Mehmood, A., Adhikari, P., Esposito, I., Elo, L. L., Zhang, F.-P., et al. (2018) Hydroxysteroid (17β) dehydrogenase 13 deficiency triggers hepatic steatosis and inflammation

in mice. *FASEB J.*, **32**, 3434–3447.

19. Pirola, C. J., Garaycoechea, M., Flichman, D., Arrese, M., San, J., Gazzi, C., Castaño, G. O. and Sookoian, S. (2019) Splice variant rs72613567 prevents worst histologic outcomes in patients with nonalcoholic fatty liver disease. *J Lipid Res*, **60**, 176–185.

20. Luukkonen, P. K., Tukiainen, T., Juuti, A., Sammalkorpi, H., Haridas, P. A. N., Niemela, O., Arola, J., Orho-Melander, M., Hakkarainen, A., Kovanen, P., et al. (2020) Hydroxysteroid 17-β dehydrogenase 13 variant increases phospholipids and protects against fibrosis in nonalcoholic fatty liver disease. *JCI Insight*, **5**, e132158.

21. Luukkonen, P. K., Juuti, A., Sammalkorpi, H., Penttilä, A., Orešič, M., Hyötyläinen, T., Arola, J., Orho-Melander, M. and Yki-Järvinen, H. (2020) MARC1 variant rs2642438 increases hepatic phosphatidylcholines and decreases severity of non-alcoholic fatty liver disease in humans. *J. Hepatol.*, https://doi.org/10.1016/j.jhep.2020.04.021.

22. Hernaez, R., McLean, J., Lazo, M., Brancati, F. L., Hirschhorn, J. N., Borecki, I. B., Harris, T. B., Nguyen, T., Kamel, I. R., Bonekamp, S., et al. (2013) Association between variants in or near PNPLA3, GCKR, and PPP1R3B with ultrasound-defined steatosis based on data from the third National Health and Nutrition Examination Survey. *Clin. Gastroenterol. Hepatol.*, **11**, 1183-1190.e2.

23. Sparsø, T., Andersen, G., Nielsen, T., Burgdorf, K. S., Gjesing, A. P., Nielsen, A. L., Albrechtsen, A., Rasmussen, S. S., Jørgensen, T., Borch-Johnsen, K., et al. (2008) The GCKR rs780094 polymorphism is associated with elevated fasting serum triacylglycerol, reduced fasting and OGTT-related insulinaemia, and reduced risk of type 2 diabetes. *Diabetologia*, **51**, 70–75.

24. Lin, Y.-C., Chang, P.-F., Chang, M.-H. and Ni, Y.-H. (2014) Genetic variants in GCKR and PNPLA3 confer susceptibility to nonalcoholic fatty liver disease in obese individuals. *Am. J. Clin. Nutr.*, **99**, 869–74.

25. Palmer, N. D., Musani, S. K., Yerges-Armstrong, L. M., Feitosa, M. F., Bielak, L. F., Hernaez, R., Kahali, B., Carr, J. J., Harris, T. B., Jhun, M. A., et al. (2013) Characterization of european ancestry nonalcoholic fatty liver disease-associated variants in individuals of african and hispanic descent. *Hepatology*, **58**, 966–975.

26. Yuan, C., Lu, L., An, B., Jin, W., Dong, Q., Xin, Y. and Xuan, S. (2015) Association Between LYPLAL1 rs12137855 Polymorphism With Ultrasound-Defined Non-Alcoholic Fatty Liver Disease in a Chinese Han Population. *Hepat. Mon.*, **15**, e33155.

27. Dold, L., Luda, C., Schwarze-Zander, C., Boesecke, C., Hansel, C., Nischalke, H. D., Lutz, P., Mohr, R., Wasmuth, J. C., Strassburg, C. P., et al. (2017) Genetic polymorphisms associated with fatty liver disease and fibrosis in HIV positive patients receiving combined antiretroviral therapy (cART). *PLoS One*, **12**, 1–13.

28. Valenti, L., Fracanzani, A. L., Bugianesi, E., Dongiovanni, P., Galmozzi, E., Vanni, E., Canavesi, E., Lattuada, E., Roviaro, G., Marchesini, G., et al. (2010) HFE Genotype, Parenchymal Iron Accumulation, and Liver Fibrosis in Patients With Nonalcoholic Fatty Liver Disease. *Gastroenterology*, **138**, 905–912.

29. Wallace, D. F. and Nathan Subramaniam, V. (2016) The global prevalence of HFE and non-HFE hemochromatosis estimated from analysis of next-generation sequencing data. *Genet. Med.*, **18**, 618–626.

30. Gallego, C. J., Burt, A., Sundaresan, A. S., Ye, Z., Shaw, C., Crosslin, D. R., Crane, P. K., Fullerton, S. M., Hansen, K., Carrell, D., et al. (2015) Penetrance of Hemochromatosis in HFE Genotypes Resulting in p.Cys282Tyr and p.His63Asp in the eMERGE Network. *Am. J. Hum. Genet.*, **97**, 512–520.

31. The UK Haemochromatosis Consortium (1997) A simple genetic test identifies 90% of UK patients with haemochromatosis. The UK Haemochromatosis Consortium. *Gut*, **41**, 841–844.

32. Canela-Xandri, O., Rawlik, K. and Tenesa, A. (2018) An atlas of genetic associations in UK Biobank. *Nat. Genet.*, **50**, 1593–1599.

33. Strnad, P., Buch, S., Hamesch, K., Fischer, J., Rosendahl, J., Schmelz, R., Brueckner, S., Brosch, M., Heimes, C. V, Woditsch, V., et al. (2018) Heterozygous carriage of the alpha1-antitrypsin Pi*Z variant increases the risk to develop liver cirrhosis. *Gut*, **68**, 1099-1107
